# Susceptibility to social influence predicts behavior on Facebook

**DOI:** 10.1371/journal.pone.0229337

**Published:** 2020-03-03

**Authors:** Sabrina Stöckli, Doris Hofer

**Affiliations:** Department of Consumer Behavior, Institute of Marketing and Management, University of Bern, Bern, Switzerland; Aalborg University, DENMARK

## Abstract

Susceptibility to social influence (SSI) has been reported as a key factor for social influence in online social networks (OSNs) such as Facebook, Instagram, and Twitter. In four online studies, we show that the personality trait of SSI, namely the susceptibility to normative influence (SNI), predicts the extent to which Facebook users comply with the behavior of others on Facebook (e.g., buying, voting, or visiting what other OSN users post). In Studies 1a and 1b, we find that SSI correlates with diverse OSN behaviors, which are the typical results of being affected by social influence. In Study 1b, we find that the perceived importance of the topic of OSN behaviors (e.g., fashion or politics) moderates the effect of SNI on OSN behavior, with a higher importance resulting in a stronger effect of SNI on OSN behavior. In Studies 2 and 3, we find that SNI predicts the extent Facebook users hypothetically “like” diverse topics on Facebook. We also find partial support for the idea that there are interactions between SNI and the Big Five personality traits (i.e., openness, conscientiousness, extraversion, agreeableness, and neuroticism) on OSN behavior. Specifically, the extent to which the Big Five personality traits of openness, agreeableness, and neuroticism predict OSN behavior depends on Facebook users’ SNI. Our studies contribute to research on the personality-based prediction of OSN behavior and help in better understanding the dynamics of social influence in OSNs, underlining the vulnerability of susceptible OSN users.

## Introduction

Social influence drives mass persuasion in online social networks (OSNs) such as Facebook, Instagram, and Twitter. Therefore, campaigners leverage social influence in OSNs to encourage people to share information [1], to adopt healthy behaviors [2], to vote for a specific politician [3], or to buy a new product [4]. Social influence in the context of OSNs can be understood as the spread of a change in OSN users’ attitudes, intentions, communication, and behaviors that are the result of the activities of others in OSNs.

One key factor for effective social influence in OSNs is users’ susceptibility to social influence (SSI) [5]. SSI can be understood as one’s tendency to change attitudes, intentions, communication, and behavior in response to others’ activities in OSNs. The traditional social influence literature implies that the personality trait of SSI has three facets: first is *susceptibility to informative influence* (SII), that is, one’s tendency to seek information from others to reduce uncertainty in a situation [6,7]. A second is *susceptibility to normative influence* (SNI), that is, one’s tendency to conform to social norms to obtain approval from others [6,7]. Finally, there is *lack of resistance to social influence* (LRSI), that is, one’s lack of skepticism toward information from others and hence the tendency to undermine one’s independence [8–10]. Having said this, a susceptible person compared with an unsusceptible person is typically more readily influenced by others—be it because of seeking information about a new product from others, because of conforming to others’ expectations of what fashion style to wear, or because of not questioning others’ posts about a political issue.

So far, there have been only a few studies testing the idea that SSI predicts to what extent users are influenced by others in OSNs [11–13]. For instance, it has been found that SSI predicts who is affected by others’ Facebook posts: susceptible Facebook users are affected by the content posted by all kinds of Facebook members, and unsusceptible Facebook users are affected only by “close” Facebook friends [11]. Likewise, SSI predicts the diffusion of word-of-mouth in OSNs. Specifically, susceptible compared with unsusceptible OSN users are more likely to pass along product information to other OSN users [12].

Interestingly, there are significant interindividual differences in how susceptible OSN users are to social influence [14,15]. To date, most of the evidence is based on social network analyses, a method using structural network metrics (e.g., based on users’ position and ties in an OSN) to identify susceptible and influential users. These analyses have demonstrated that susceptible users are key to the spread of information or behavior in OSNs [5,14,16]. By inferring OSN users’ SSI from structural network metrics, however, this method risks confusing the personality trait of SSI with some specific consequences of SSI, namely users’ structural position or ties in the OSN. To separate SSI as a personality trait from what may be only structural consequences of SSI, it seems promising to measure SSI using psychometric scales and investigate its relationship to behavior that determines OSN users’ position and ties in OSNs.

From a psychological perspective, little is known about interindividual differences in SSI in OSNs. Drawing from the traditional social influence literature that focuses on the offline context, it is likely that SSI is a stable personality trait that also determines behavior in OSNs [17]. For instance, it is likely that a susceptible (vs. unsusceptible) OSN user is comparably more influenced by other OSN users’ activities, such as political posts or sharing holiday pictures. Although SSI is a personality characteristic considered to be stable across different topics (e.g., politics or consumption), we do not rule out contextual differences. For example, a susceptible OSN user might be influenced by other OSN users to a higher extent if the topic of the OSN behavior (e.g., politics vs. fashion) is perceived as being important [18–20]. To the best of our knowledge, it remains unexplored whether the effect of SSI on OSN behavior depends on the perceived importance of the topic of OSN behavior.

Despite the significance of OSN users’ SSI, research on the application of psychometric scales for the prediction of OSNs (e.g., for advertising purposes) does not consider the SSI personality trait. Indeed, the research has so far focused on other personality traits, namely the Big Five personality traits (i.e., openness, conscientiousness, extraversion, agreeableness, and neuroticism) to predict OSN behavior ([21], and for an exception, see [22]). There is increasing evidence showing that the Big Five personality traits predict diverse digital footprints, such as Facebook likes. For instance, people who score high (vs. low) on extraversion are more likely to give a Facebook like to “socializing” [23,24]. Regarding the predictive power of personality traits for OSN behavior, it seems informative to refer to a meta-analysis on the studies that have examined the association between the Big Five personality traits and OSN behavior; the meta-analysis shows that the strength of the relationship between the Big Five personality traits and OSN behavior is between *r* = 0.29 and *r* = 0.40 (*r* = 0.29 for agreeableness, *r* = 0.33 for neuroticism, *r* = 0.35 for conscientiousness, *r* = 0.39 for openness, and *r* = 0.40 for extraversion). This effect size range seems to be in accordance with the “personality coefficient,” that is, the typical strength of the relationship between personality traits and behavior (i.e., *r* = 0.30–0.40) [21].

Regarding the aim of improving the understanding and prediction of OSN behavior based on personality traits, it seems promising to examine the Big Five personality traits in combination with SSI. Starting from the premise that SSI predicts to what extent OSN users are influenced by others, we expect that the Big Five personality traits determine OSN behavior to a larger extent when SSI is high (vs. low). For example, we expect that users who score high on openness have a stronger tendency to like posts with a high-openness content on Facebook when they are also susceptible (vs. unsusceptible) to the posts and likes of others. In other words, we expect SSI to moderate the effect of the Big Five personality traits on OSN behavior, such as Facebook liking behavior.

Currently, a critical question—not only for researchers but also for OSN services and campaigners—is how to most accurately predict OSN behavior. OSN services and campaigners want to optimize psychological targeting. To predict OSN behavior, researchers, OSN services, and campaigners must mine for the digital footprints that are most indicative of the user personality traits that can ensure effective prediction of social influence, that is, persuasion in OSNs [25,26]. SSI is certainly one of those user personality traits. Having said this, accurate predictions of OSN behavior seem not only to be beneficial (e.g., fostering healthy behaviors) but also harmful (e.g., being invasive; see [20]). To date, we know little about the scope of the consequences of psychological targeting, that is, using the relationship between personality and OSN behavior for effective mass persuasion in OSNs. Thus, independent and publicly available research on this topic is required and should serve as a basis for political and legal measures against the harmful consequences of psychological targeting in OSNs.

In the present research, we address the idea that SSI as a personality trait can explain OSN behavior. To examine this, we test three hypotheses: First, we test if and to what extent the different facets of SSI correlate with diverse OSN behaviors (Studies 1a and 1b). Second, we test whether the effect of SSI on OSN behavior also depends on the topic’s importance; we expect the effect of SSI on OSN behavior to be more (or only) present when the behavior concerns a topic that is important to OSN users (Study 1b). Third, we test whether SSI predicts a user’s liking behavior in OSNs; thereby, we also explore whether SSI moderates the effect of the Big Five personality traits on OSN behavior. Here, we expect that susceptible compared with unsusceptible OSN users will be more interested in OSN content that corresponds to their Big Five personality profile when this content comes from the posts of other OSN users (Studies 2 and 3).

## Study 1a

### Method

#### Sample

Facebook users (*N* = 118, 70% female, 30% male, *M*_*age*_ = 26.6, *SD*_*age*_ = 6.9) were recruited through Facebook by the first author and student assistants to participate in a 10–15 minute online survey about “social media use.” The survey was in German and we only addressed German-speaking Facebook users. We only included participants who fully completed the survey. Note that we set a minimum age of 14 to participate in the survey. Because our study does not involve risk to the participants, the ethics committee of our university approved this procedure (with the minimum age) based on the relevant federal Act on Research involving Human Beings. As an incentive, the participants were offered a place in a prize drawing for a voucher worth 100 Swiss Francs. The preregistration, material, and code of the current study can be found on the Open Science Framework (OSF; https://osf.io/e5zsn/). Note that the Ethics Committee of the Faculty of Business Administration, Economics and Social Sciences of the University of Bern approved all of the present studies (project serial number: 022019). In all studies, participants provided informed consent to participate by clicking "ok" in the online survey, and their data were anonymized.

Regarding the sample size calculation, we assumed that an *r* of 0.20 would be reasonable (see [22]). We ran a power analysis (R pwr package version 1.2–2; [27]), here assuming *α* = 0.05 and *β* = 0.20. Based on this, we defined the sample size as 200. Because it was difficult to attain the sample size, we decided to stop data collection after 60 days (118 participants were reached). Because of the exploratory character of Study 1a, we considered stopping data collection at this point as reasonable. We did not analyze our data before the 60 days had passed.

#### Design and procedure

In the correlational online survey, the participants first indicated the frequency of 15 OSN behaviors interpreted as evidence of being influenced by others in OSNs. Second, they filled in a set of psychometric scales that captured the different facets of SSI. Finally, the participants provided their sociodemographic information.

#### Material

*Online social network behaviors*. We defined 15 different OSN behaviors that are evidence of being influenced by others in OSNs. These OSN behaviors encompassed various topics, such as politics, fashion, music, or traveling. A list of all 15 OSN behaviors can be found in the S1 Appendix Table 1. The participants were asked to indicate the frequency of showing these OSN behaviors on a 5-point Likert scale ranging from 1 (*never*) to 5 (*always*). An example of one of the 15 OSN behaviors is the following: “I have read other Facebook users’ posts on political content.”

Note that OSN behaviors cover different degrees of social influence. We used OSN behaviors with variances in terms of the degree of social influence to account for the fact that social influence can result in small and pronounced changes in one’s attitudes, intentions, communication, and behaviors. For example, we used the OSN behavior “reading other OSN users’ posts on political content” (small influence) and “purchasing products that I have become aware of through other OSN users’ posts” (pronounced influence).

*Susceptibility to social influence*. To measure the participants’ SSI, we used different psychometric scales that can capture the various facets of SSI. To identify suitable SSI scales, we conducted a literature review (for details on the procedure of the literature review, see S1 Appendix Table 2). The following seven psychometric scales qualified as capturing the different facets of SSI: First, we used the susceptibility-to-informative-influence scale [6] and the information-seeking scale [28]. Both capture the tendency to seek information from others (SII facet of SSI). Further, we used the susceptibility-to-normative-influence scale [6]; this scale captures the tendency to comply with social norms (SNI facet of SSI). For explorative purposes, we used three additional scales that can capture the constructs associated with SNI: the attention-to-social-comparison-information scale (i.e., tendency to pay attention to reactions to one’s behavior; [29], the public-self-consciousness scale (i.e., the tendency to be aware of the impression one makes on others; [30], and the need-for-consistency scale (need to appear consistent to others; [31]). Finally, we considered the lack-of-skepticism scale [32], which captures the tendency to be skeptical of information from others (LRSI facet of SSI).

For all of the psychometric scales, the participants indicated the extent to which scale items were true for them, ranging from 1 (*never true*) to 5 (*always true*). The S1 Appendix provides detailed descriptions and the items of all the SSI scales (S1 Appendix Tables 2 and 3). The reliabilities (Cronbach’s α) of the SSI scales were between 0.71 and 0.91 (for an overview of all Cronbach’s α values, see S1 Appendix Table 4).

### Results

#### Susceptibility to normative influence correlates with online social network behavior

To test if and to what extent SSI correlates with OSN behavior, we conducted a regression analysis. For this purpose, we used the mean of all 15 OSN behaviors as the response variable (*M* = 1.97, *SD* = 0.59) and the seven SSI scales as the predictor variables. Details concerning the overall significance of the regression, as well as details concerning the parameter estimates, are provided in Table 1. As Table 1 depicts, the susceptibility-to-normative-influence scale best predicts OSN behavior. In the S1 Appendix, we provide a detailed overview of the separate correlations of the seven SSI scales and the 15 OSN behaviors. S1 Appendix Fig 1A depicts a heat map of all the correlations, and S1 Appendix Table 4A provides Pearson correlations and BCa bootstrap CIs.

**Table 1:** Testing whether SSI scales predict OSN behavior.

	*Β*	SE	95% CI for *B*	*t*	*p*
Response variable: OSN behavior					
(Intercept)	1.04	0.28	[0.50, 1.59]	3.77	< 0.001
Susceptibility-to-informative influence scale	0.13	0.08	[-0.04, 0.30]	1.50	0.137
Information-seeking scale	-0.10	0.08	[-0.27, 0.07]	1.19	0.238
Susceptibility-to-normative-influence scale	0.23	0.11	[0.01, 0.46]	2.06	< 0.05
Attention-to-social-comparison-information scale	0.01	0.10	[-0.19, 0.22]	0.14	0.886
Public-self-consciousness scale	0.11	0.09	[-0.07, 0.29]	1.23	0.220
Need-for-consistency scale	0.07	0.08	[-0.09, 0.23]	0.91	0.363
Lack-of-skepticism scale	-0.08	0.10	[-0.27, 0.12]	0.79	0.430

Overall significance of the model: *F*(7, 110) = 3.85, *p* < 0.001, adjusted *R*^*2*^ = 0.15.

In Study 1b, we aimed to replicate Study 1a but in a slightly modified way. We ran Study 1b for three reasons: First, we aimed to replicate Study 1a to assess the study’s validity, reliability, and generalizability. In particular, we wanted to strengthen the results of our first exploration, namely that SNI is the most important facet of SSI in terms of predicting OSN behavior. Second, we aimed to test the correlation of OSN behaviors with an additional scale (need-for-uniqueness scale) that might be related to SSI; therefore, we added this scale to the survey from Study 1a. Third, we aimed to test whether the effect of SSI on OSN behavior is more (or only) present when the behavior concerns a topic that is important to OSN users. Therefore, we queried the importance of the topics for the respective OSN behaviors.

## Study 1b

### Method

#### Sample

Facebook users (*N* = 372, 63% female, 37% male, *M*_*age*_ = 25.0, *SD*_*age*_ = 5.8) were recruited online through Facebook by the first author and student assistants to participate in a 10–15 minute survey about “social media use.” The survey was in German and we only addressed German-speaking Facebook users. Furthermore, we only included participants who fully completed the survey. As in Study 1a, only people older than 14 years of age were allowed to participate in the survey. As an incentive, the participants were given the chance to take part in a prize drawing for one of three vouchers worth 100 Swiss Francs. The preregistration, material, and code for this study can be found on the OSF website (https://osf.io/kea3r/).

Regarding the sample size calculation, we ran a power analysis. For simplicity reasons, we used the pwr.f2.test function for general linear models (R pwr package version 1.2–2; [27]). We ran analyses for small and medium effect sizes (i.e., for *f*^*2*^ = 0.02 and *f*^*2*^ = 0.15), assuming *α* = 0.05 and *β* = 0.20. Based on this, we defined the sample size as 400. Because it was difficult to attain the sample size, we decided to stop data collection after 90 days (372 participants were reached). We did not analyze our data before the 90 days had passed.

#### Design and procedure

As in Study 1a, we conducted a correlational online survey in which the participants first indicated the frequency of 15 OSN behaviors interpreted as evidence of being influenced by others in OSNs. Second, the participants indicated how important the topics of these OSN behaviors (e.g., politics or fashion) were to them. Third, the participants filled in the SSI scales and then provided their sociodemographic information.

#### Material

*Online social network behaviors and susceptibility to social influence*. The material for Study 1b was the same as for Study 1a. That is, we used the same 15 OSN behaviors and the same SSI scales (an overview of all OSN behaviors and SSI scales can be found in S1 Appendix 1–3).

Note that in addition to the SSI scales in Study 1a, we also considered the need-for-uniqueness scale [33], which captures the need to appear unique to others (for more details and the items of the scale, see S1 Appendix 2). We considered this additional psychometric scale for exploratory purposes. Specifically, after Study 1a, we believed that OSN users who have a strong desire for uniqueness would be comparably unlikely to be influenced by other users in OSNs. Hence, we wanted to explore whether there is a negative correlation between the need for uniqueness and OSN behavior that results from social influence.

For all of the psychometric scales, the participants indicated the extent to which the scale items were true for them, ranging from 1 (*never true*) to 5 (*always true*). The reliabilities (Cronbach’s α) of the SSI scales were between 0.72 and 0.89 (for an overview of all Cronbach’s α values, see S1 Appendix Table 5).

*Topic importance*. To test whether the effect of SSI on OSN behavior is more (or only) present when the behavior concerns a topic that is important to the OSN users, we measured the importance of the topics of the OSN behaviors. Note that asking for the topic importance of the 15 OSN behaviors seemed to be meaningful only for nine OSN behaviors. Specifically, we asked for the importance of the following topics: politics, fashion, traveling, leisure, food, music, news, brands, and charity (see S1 Appendix Table 1). The topic “politics” was asked, for example, because of the OSN behavior “I have read other Facebook users’ posts on political content.” One OSN behavior where it did not seem meaningful to ask about its importance was, for example, “I have forwarded/shared posts from other Facebook users.” Forwarding posts does not represent a meaningful topic such as, for example, with politics and fashion, and asking how important it is to forward posts sounds somewhat awkward. The participants indicated the personal importance of the nine selected topics on a 5-point Likert scale ranging from 1 (*unimportant*) to 5 (*important*).

### Results

#### Susceptibility to normative influence correlates with online social network behavior

To test if and to what extent SSI correlates with OSN behavior, we conducted a regression analysis. As in Study 1a, we used the mean of all 15 OSN behaviors as the response variable (*M* = 1.76, *SD* = 0.62) and the eight SSI scales as the predictor variables. Details concerning the overall significance of the regression model, as well as details concerning the parameter estimates, are provided in Table 2. In line with Study 1a, Table 2 depicts that the susceptibility-to-normative-influence scale was the best predictor of OSN behavior—followed by the lack-of-skepticism and need-for-uniqueness scales—underlining that SNI seems to be the most important facet of SSI in predicting OSN behavior. In the S1 Appendix, we additionally provide a detailed overview of the separate correlations of the eight SSI scales and the 15 OSN behaviors (see S1 Appendix Fig 1B for a heat map with all the correlations and S1 Appendix Table 5 for all Pearson correlations).

**Table 2:** Testing whether SSI scales predict OSN behavior.

	*Β*	SE	95% CI for *B*	*t*	*p*
Response variable: OSN behavior					
(Intercept)	0.91	0.22	[0.48, 1.35]	4.12	< 0.001
Susceptibility-to-informative-influence scale	0.00	0.05	[-0.10, 0.09]	0.10	0.922
Information-seeking scale	0.08	0.05	[-0.01, 0.18]	1.73	0.084
Susceptibility-to-normative-influence scale	0.20	0.06	[0.08, 0.33]	3.14	< 0.01
Attention-to-social-comparison-information scale	0.02	0.07	[-0.11, 0.16]	0.32	0.745
Public-self-consciousness scale	-0.08	0.06	[-0.19, 0.04]	1.36	0.176
Need-for-consistency scale	0.00	0.05	[-0.10, 0.04]	0.02	0.985
Lack-of-skepticism scale	0.16	0.05	[0.06, 0.27]	3.40	< 0.05
Need-for-uniqueness scale	0.09	0.04	[0.01, 0.18]	2.10	< 0.05

Overall significance of the model: *F*(8, 363) = 8.28, *p* < 0.001, adjusted *R*^*2*^ = 0.14.

Overall, Studies 1a and 1b support the idea that SSI as a personality trait explains OSN behavior. Three points should be noted here: First, the susceptibility-to-normative-influence scale correlates the most with OSN behavior in both studies. Second, other psychometric scales, such as the need-for-consistency scale, hardly correlate with OSN behavior. Third, the correlations of some SSI scales with OSN behavior seem to differ across the two studies. Specifically, the lack-of-skepticism and the need-for-uniqueness scales correlate significantly with OSN behavior in Study 1b but not in Study 1a. Because the susceptibility-to-normative-influence scale correlates the most with OSN behavior in both studies, we will subsequently focus our analyses on the SNI facet of SSI.

#### Topic importance moderates the effect of susceptibility to normative influence on online social network behavior

Based on the results from Studies 1a and 1b, we only used the SNI facet instead of multiple SSI facets to test whether the effect of SSI on OSN is more present when the topic of OSN behavior is important. To this end, we conducted a repeated measure linear mixed effects regression analysis. Using the lmer function of the R lme4 package (version 1.1–21, [34]), we fit the following model: As the response variable, we used the indicated frequency of showing the nine different OSN behaviors (repeated measure). As the fixed factors, we used SNI, topic importance, and their interaction. For SNI, we used participants’ scale mean scores of the susceptibility-to-normative-influence scale. For topic importance, we used the importance values that the participants indicated separately for all nine topics. Note that we mean centered both of the fixed factors. The random structure was specified by entering a random intercept for both the participants and the different topics. We included the participants and topics as crossed random effects to adjust for the intercepts for variance conditional to the participants and topics (see [35,36]). The model was estimated using REML. The model and computational details are provided in S1 Appendix 6.

As the results show, there was an interaction between SNI and topic importance. An analysis of deviance (Type II Wald F tests with Kenward-Roger df) was computed with the ANOVA function of the R car package (version 2.0–15; [37]) to determine the contribution of each predictor to the model fit (SNI*topic importance: *F* = 20.08, *df* = 1, *df*.*res* = 3252, *p* < 0.001; SSI: *F* = 41.08, *df* = 1, *df*.*res* = 371, *p* < 0.001; topic importance: *F* = 296.852, *df* = 1, *df*.*res* = 3270, *p* < 0.001). As Table 3 depicts, the effect of SNI on OSN behavior was moderated by the perceived topic importance (*B* = 0.06). That is, the effect of SNI on OSN behavior was stronger when the OSN behavior concerned a topic that was important to OSN users. Further, SNI (*B* = 0.27) and topic importance (*B* = 0.17) affected OSN behavior. Taken together, this supports the idea that SSI—or, more specifically, SNI—determines OSN behavior and that this effect depends on how important the topic of the OSN behavior is to the OSN users.

**Table 3:** Parameter estimates for testing topic importance as a moderator of the effect of SNI on OSN behavior.

Parameters				
Fixed effects	*B*	*SE*	*t*	*p*
(Intercept)	1.78	0.14	13.09	<0.001
SNI	0.27	0.04	6.21	**<0.001**
Topic importance	0.17	0.01	17.36	**<0.001**
SNI*topic importance	0.06	0.01	4.48	**<0.001**
Random effects	*Variance*	*SD*		
Participants (intercept)	0.26	0.51		
Topics (intercept)	0.16	0.40		

The linear mixed effects regression analysis was run on 3,348 observations with 372 participants. The unconditional ICCs are as follows: ICC_participants_ = 0.28, ICC_OSN behaviors_ = 0.20, ICC_participants+OSN behaviors_ = 0.47. Marginal *R*^*2*^ = 0.12 (i.e., variance of the fixed effects), and conditional *R*^*2*^ = 0.51 (i.e., variance of the fixed and random effects).

On the repeated measure response variable (OSN behavior), higher values represent a higher frequency of engaging in social influence. The table depicts the fixed effects coefficients and standard errors (for SNI, topic importance, and their interaction), as well as the random effects variance and standard deviation (for the participants and topics). Note that bold *p* values are also significant after Bonferroni correction.

Given that Studies 1a and 1b support the idea that SSI, namely the SNI facet of SSI, predicts diverse self-reported OSN behaviors, we aimed to test this effect of SNI on the concrete behavior of liking Facebook topics. Furthermore, we wanted to explore whether SSI moderates the effect of the Big Five personality traits on OSN behavior. Here, we expected that OSN users would be more interested in OSN content (e.g., posts or comments from other OSN users) that corresponds to their Big Five personality profile when they are susceptible compared with unsusceptible to social influence.

## Study 2

### Method

#### Sample

We recruited U.S. participants through Amazon MTurk to participate in a 10 minute correlational online survey on “social media use” (*N* = 298, 60% female, 40% male, *M*_*age*_ = 35.3, *SD*_*age*_ = 9.7). We asked the participants to only take part in the study if they had a Facebook account and used it at least five times a month. The survey was conducted in English. The participants received $2.50 each for taking part in the study. The preregistration, material, and code of this study can be found on the OSF website (https://osf.io/yuenm/).

Regarding the sample size calculation, we ran a power analysis. For simplicity reasons, we used the pwr.f2.test function for general linear models (R pwr package version 1.2–2; [27]). We ran the analyses for small and medium effect sizes (i.e., for *f*^*2*^ = 0.02 and *f*^*2*^ = 0.15), assuming *α* = 0.05 and *β* = 0.20, which defined the sample size as 300 participants. Note that we had to exclude 24 participants who failed the attention check (298 participants remained after this exclusion).

#### Design and procedure

We conducted an online survey in which the participants first reported their hypothetical Facebook liking behavior. Specifically, the participants indicated the likelihood of clicking “like” for popular Facebook topics given that one or more of their Facebook friends would have “liked” it. Finally, the participants filled out the questionnaires, which captured their SNI, their extraversion, and their openness.

#### Material

*Facebook liking behavior*. To test whether SNI predicts OSN behavior combined with the Big Five personality traits, we measured Facebook liking behavior as our response variable (OSN behavior). We assumed someone “liking” Facebook topics after realizing that one or more of that individual’s Facebook friends have “liked” these Facebook topics can be interpreted as evidence of social influence.

We showed 20 diverse Facebook topics to the participants and asked them to rate the likelihood that they would like these Facebook topics after having noticed that their Facebook friends had liked the same topics (from 0 *(not likely)* to 100 *(very likely)*; or *no answer*). Note that the participants were instructed that if they had already liked a certain Facebook topic, they should indicate that they would be very likely (=100) to like this topic. We used 20 Facebook topics that research has indicated are highly related to one of the two Big Five personality traits: openness or extraversion. Specifically, we used four sets of Facebook topics (five Facebook topics each) that were either indicative of low openness (e.g., I don’t read), high openness (e.g., Oscar Wilde), low extraversion (e.g., programming), or high extraversion (e.g., socializing). See S2 Appendix Table 1 for more details of the four sets of Facebook topics.

*Susceptibility to normative influence*, *openness*, *and extraversion*. We used the susceptibility-to-normative-influence scale to capture the SNI facet of SSI ([6], for the description and items, see S1 Appendix 2 and 3). The reliability (Cronbach’s α) of the scale was 0.93. As in Studies 1a and 1b, the participants indicated for all scales the extent to which the scale items were true for them on a 5-point Likert scale (from 1 (*never true*) to 5 (*always true*)).

To capture openness and extraversion, we used the short International Personality Item Pool questionnaire ([38]; see S2 Appendix Table 2). The reliability (Cronbach’s α) was 0.83 for openness and 0.84 for extraversion.

### Results

**Fig 1:**
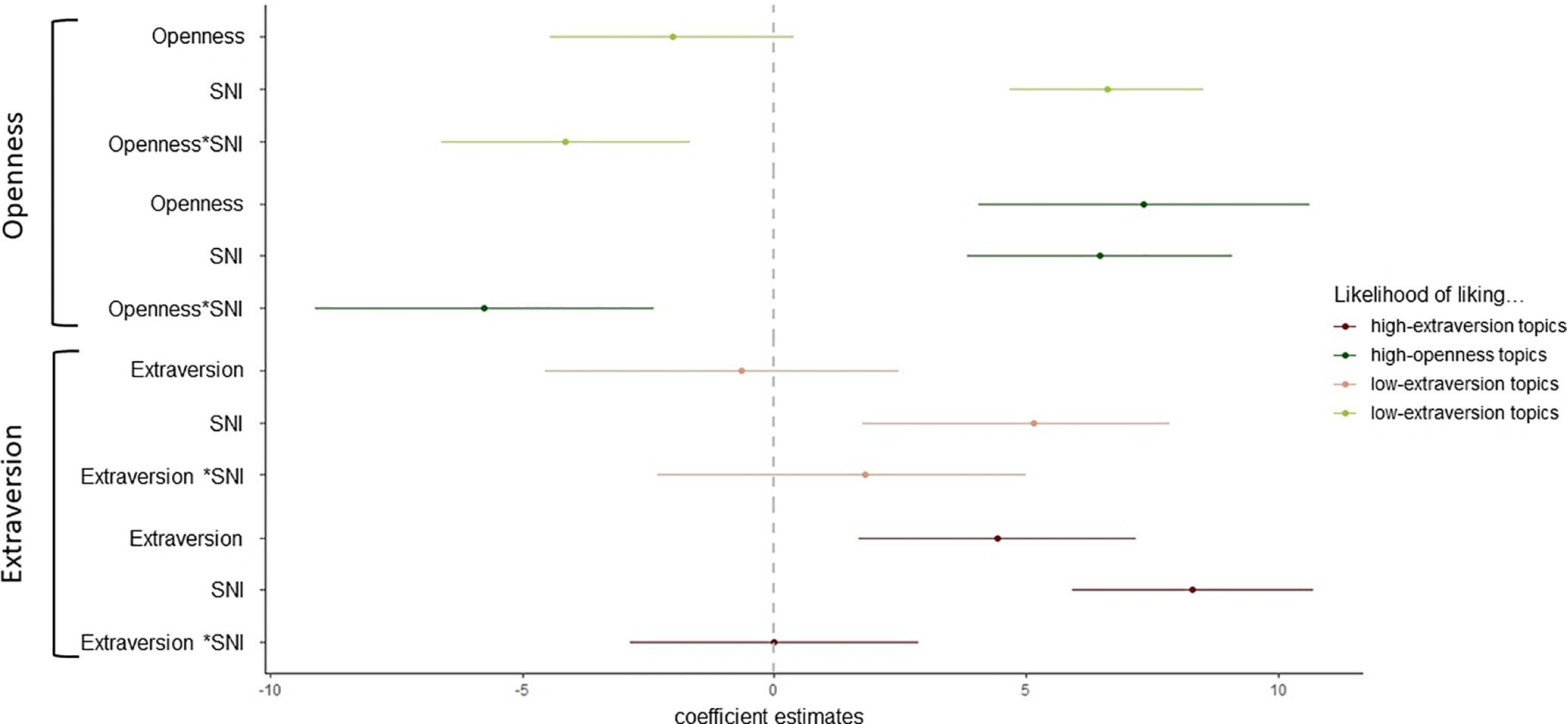
Coefficient estimates for the regression analyses with SNI and openness/extraversion on Facebook liking behavior. An overview of the coefficient estimates with the CIs for the four regression analyses is as follows: (1) likelihood of liking low-openness topics = Openness*SNI (2) likelihood of liking high-openness topics = Openness*SNI (3) likelihood of liking low-extraversion topics = Extraversion*SNI (4) likelihood of liking high-extraversion topics = Extraversion*SNI. Regarding the aim of Study 2, it should be noted that all four regression analyses revealed a conditional effect of SNI, substantiating that SNI drives OSN users to like what others in OSNs like. Furthermore, the depicted coefficient estimates for the interactions show that there is only partial support for the idea that SNI moderates the effect of the Big Five personality traits on OSN behavior.

To test whether SNI, the Big Five personality traits (openness and extraversion), and their interactions predict to what extent people (hypothetically) like Facebook topics that are related to the respective Big Five personality trait, we conducted four regression analyses. An overview of the four regression analyses can be found in Fig 1. A table with the details concerning the overall significance of the models, as well as details concerning the parameter estimates for the interactions and conditional effects, is provided in S2 Appendix Table 3.

#### Susceptibility to normative influence predicts liking openness topics on Facebook

For the first regression analysis, we used the mean of the likelihood rating of liking the low-openness Facebook topics as the response variable (*M* = 10.32, *SD* = 15.74). SNI, openness, and their interaction were used as the predictor variables (mean centered). As Fig 1 depicts, there was an interaction between SNI and openness. A visual inspection of the interaction using the Johnson–Neyman technique (see S2 Appendix Figs 4–5) shows that higher levels of SNI (0.15 *SD* or further above the mean) were associated with a stronger negative effect of openness on liking low-openness topics. Specifically, only for susceptible but not for unsusceptible OSN users, low (vs. high) openness increased the likelihood of liking low-openness topics. Furthermore, there was a conditional positive effect of SNI. This can be interpreted as a positive effect of SNI when openness is at its mean, or in other words, that the higher an “average openness user” scored on SNI, the more likely she or he has liked low-openness topics. There was no conditional effect of openness (see Fig 1). Note that an ICC(2,*k*) of 0.73 shows there was an acceptable degree of agreement with which the participants indicated the likelihood of liking the five low-openness topics (*F*(297, 1188) = 3.9, *p* < 0.001, 95% CI [0.68, 0.78]) (ICC function of R DescTool package version 0.00.19; see [39, 40]). Hence, it seemed justified to build a mean score of the likelihood rating of the five Facebook topics.

For the second regression analysis, we used the mean of the likelihood rating of liking the high-openness Facebook topics as the response variable (*M* = 18.55, *SD* = 20.67). SNI, openness, and their interaction were used as the predictor variables. The results reveal that there was an interaction between SNI and openness (see Fig 1). A visual inspection of the interaction with the Johnson–Neyman technique (see S2 Appendix Figs 6–7) shows that lower levels of SSI (0.7 *SD* above the mean or lower) were associated with a stronger positive effect of openness on liking high-openness topics. Specifically, only for unsusceptible but not for susceptible OSN users, high (vs. low) openness increased the likelihood of liking high-openness topics. In addition, there was a conditional positive effect of SNI. This means that the higher an “average openness user” scored on SNI, the more likely she or he has liked high-openness topics. Further, there was a conditional positive effect of openness. This means that the higher an “average susceptible user” scored on openness, the more likely she or he has liked high-openness topics (see Fig 1). Note that there was a good degree of agreement with which the participants indicated the likelihood of liking the five high-openness topics: ICC(2,*k*) = 0.80, *F*(297, 1188) = 5.3, *p* < 0.001, 95% CI [0.76, 0.84]).

#### Susceptibility to normative influence predicts liking extraversion topics on Facebook

For the third regression analysis, we used the mean of the likelihood rating of liking low-extraversion topics as the response variable (*M* = 28.7, *SD* = 23.54). SNI, extraversion, and their interaction were used as the predictor variables (mean centered). The results reveal that there was no interaction. Although there was also no conditional effect of extraversion, we found a conditional positive effect of SNI (see Fig 1). Note that there was an acceptable degree of agreement with which a participant indicated the likelihood of liking the five low-extraversion topics: ICC(2,*k*) = 0.75, *F*(297, 1188) = 4.8, *p* < 0.001, 95% CI [0.67, 0.81]).

For the fourth regression analysis, we used the mean of the likelihood rating of liking high-extraversion topics as the response variable (*M* = 18.75, *SD* = 19.90). SNI, extraversion, and their interaction were used as the predictor variables. The results reveal that there was no interaction between SNI and extraversion. There were, however, conditional positive effects of SSI and extraversion (see Fig 1). Note that there was a good degree of agreement with which the participants indicated the likelihood of liking the five high-extraversion topics: ICC(2,*k*) = 0.77, *F*(297, 1188) = 5.1, *p* < 0.001, 95% CI [0.69, 0.82]).

Overall, the results support the idea that SSI, namely SNI, predicts OSN behavior: all four regression analyses revealed that susceptible compared with unsusceptible users were more likely to have their Facebook liking behavior influenced by others in OSNs (conditional effect of SSI). Regarding the idea that SNI moderates the effect of the Big Five personality traits on OSN behavior, we found mixed results. In line with our idea, susceptible but not unsusceptible users who scored low (vs. high) in openness were more likely to like low-openness topics. Unsusceptible users with high vs. low scores in openness did not differ in their likelihood to like low-openness topics. In contrast to our idea, the effect that high (vs. low) openness is related to a higher likelihood of liking high-openness topics was only found for unsusceptible, not for susceptible, users. Although there was the expected effect that susceptible (vs. unsusceptible) users were more likely to like high-openness topics, there was no difference between susceptible users who scores low in openness and susceptible users who scored high in openness. Further, we found no support for the idea that SNI moderates the effect of extraversion on OSN behavior (i.e., liking extraversion topics).

Given that Study 2 was limited to the Big Five personality traits of openness and extraversion, we ran an extended version of Study 2 to test whether SNI moderates the effect of all of the Big Five personality traits on Facebook liking behavior. Note that we also aimed to replicate Study 2 with a larger sample size for increased validity, reliability, and generalizability.

## Study 3

### Method

#### Sample

We recruited U.S. participants through Amazon MTurk to participate in a 10–15 minute correlational online survey on “social media use” (*N* = 544, 56% female, 43% male, 1% nonbinary, *M*_*age*_ = 35.8, *SD*_*age*_ = 10.5). We asked the participants to participate only in the study if they had a Facebook account and used it at least five times a month. The survey was conducted in English, and the participants received $2.50 for taking part in the study. The preregistration, material, and code of this study can be found on the OSF website (https://osf.io/adhgy/).

Regarding the sample size calculation, we wanted to make sure we could detect even a small effect; hence, we assumed *f*^*2*^ = 0.02. Based on a power analysis (R pwr package version 1.2–2; [27]), here assuming *α* = 0.05 and *β* = 0.20, we defined the sample size as 550. Note that we had to exclude 15 participants because of a failed attention check. We considered the resulting sample of 544 participants to be suitable.

#### Design and procedure

We conducted an online survey in which the participants first reported their Facebook liking behavior. Similar to Study 2, the participants indicated the likelihood of clicking “like” for popular Facebook topics given the fact that one or more of their Facebook friends would have “liked” it. Afterwards, the participants filled out questionnaires capturing their SNI and Big Five personality traits.

#### Material

*Facebook liking behavior*. As in Study 2, hypothetical Facebook liking behavior was measured as the response variable. Again, we did this to test whether SNI predicts OSN behavior, namely whether OSN users are more likely to give “likes” to Facebook topics if one or more of the individual’s Facebook friends have “liked” it. Similar to Study 2, we gave the participants a list of diverse Facebook topics and asked them to rate the likelihood that they would like these Facebook topics after having noticed that their Facebook friends had liked the same topics (from 0 *(not likely)* to 100 *(very likely)*; or *no answer*). If they had already liked a certain Facebook topic, the participants were instructed to indicate that they would be very likely (=100) to like this topic. We used Facebook topics from the literature on predicting OSN behavior based on digital footprints, here finding what would be highly indicative for both low or high expressions of the Big Five personality traits [24]. Specifically, we used 10 sets of Facebook topics (10 Facebook topics per set) that were either indicative of low openness (e.g., I don’t read), high openness (e.g., Oscar Wilde), low conscientiousness (e.g., Wes Anderson), high conscientiousness (e.g., accounting), low extraversion (e.g., programming), high extraversion (e.g., socializing), low agreeableness (e.g., knives), high agreeableness (e.g., Christianity), low neuroticism (e.g., soccer), or high neuroticism (e.g., so, so happy). See S3 Appendix Table 1 for a full list of the 10 sets of Facebook topics (100 Facebook topics in total).

*Susceptibility to normative influence and the Big Five personality traits*. Again, we used the susceptibility-to-normative-influence scale ([6], see S1 Appendix 2 and 3 for a description and the items) to capture the SNI facet of SSI. As in Studies 1a to 2, the participants indicated the extent to which the scale items were true for them on a 5-point Likert scale (from 1 (*never true*) to 5 (*always true*)). The reliability (Cronbach’s α) of the susceptibility-to-normative-influence scale was 0.94.

To capture the Big Five personality traits, we used the short International Personality Item Pool questionnaire ([38]; see S2 Appendix Table 2 for items). The reliability (Cronbach’s α) was 0.77 for openness, 0.85 for conscientiousness, 0.88 for extraversion, 0.82 for agreeableness, and 0.88 for neuroticism.

### Results

**Fig 2:**
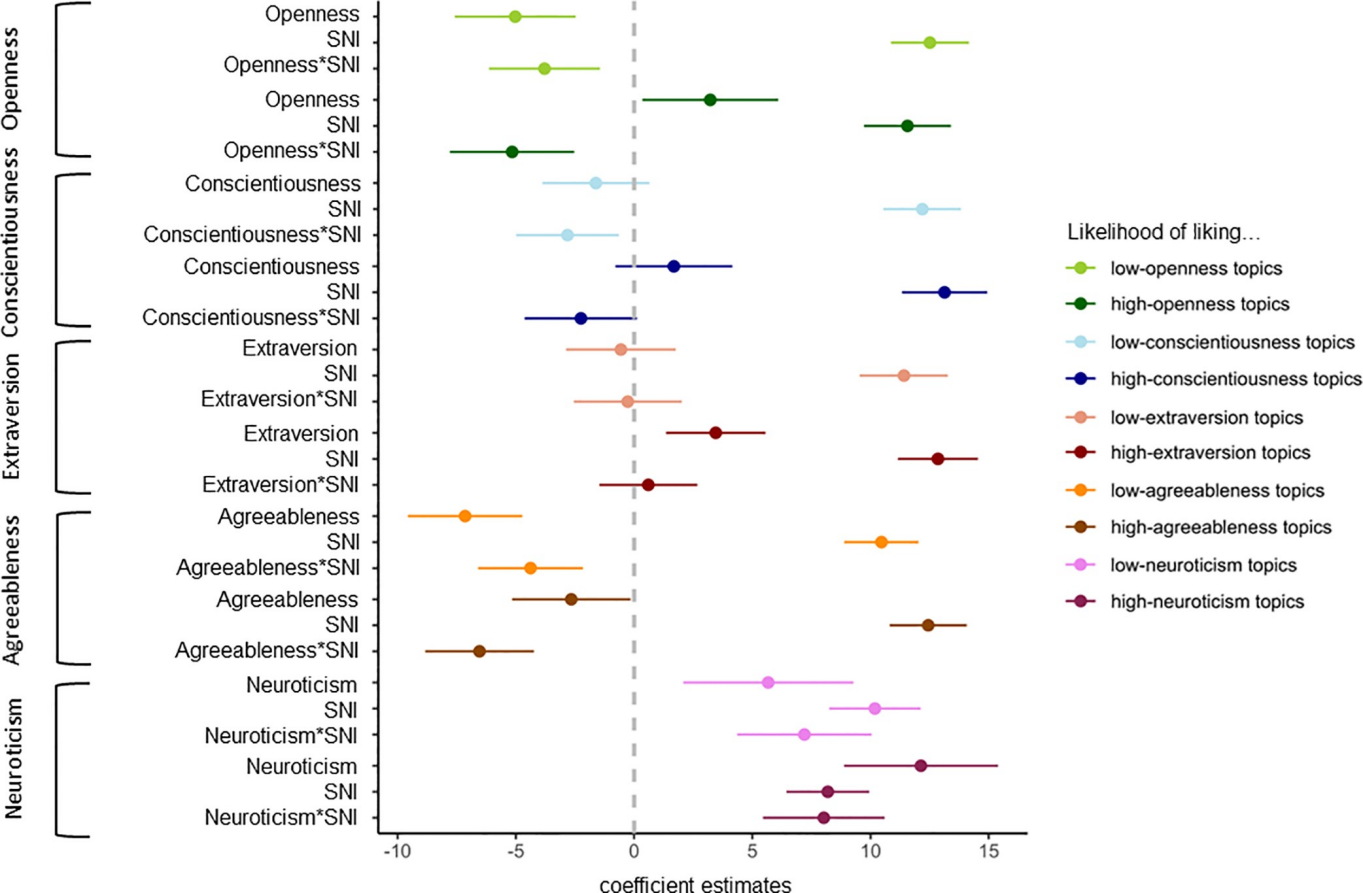
Coefficient estimates for the regression analyses with SNI and the Big Five personality traits on Facebook liking behavior. An overview of the coefficient estimates with the CIs for the 10 regression analyses is as follows: (1) likelihood of liking low-openness topics = Openness*SNI (2) likelihood of liking high-openness topics = Openness*SNI (3) likelihood of liking low-conscientiousness topics = Conscientiousness *SNI (4) likelihood of liking high-conscientiousness topics = Conscientiousness *SNI (5) likelihood of liking low-extraversion topics = Extraversion*SNI (6) likelihood of liking high-extraversion topics = Extraversion*SNI (7) likelihood of liking low-agreeableness topics = Agreeableness*SNI (8) likelihood of liking high-agreeableness topics = Agreeableness*SNI (9) likelihood of liking low-neuroticism topics = Neuroticism*SNI (10) likelihood of liking high-neuroticism topics = Neuroticism*SNI. Regarding the aim of Study 3, it should be noted that all 10 regression analyses revealed a conditional effect of SNI, substantiating that SNI drives OSN users to like what others in OSNs like. Furthermore, the depicted coefficient estimates for the interactions show that there is only partial support for the idea that SSI moderates the effect of the Big Five personality traits on OSN behavior.

To test whether SNI, the Big Five personality traits, and their interactions could predict to what extent people like Facebook topics related to the respective Big Five personality trait, we conducted 10 regression analyses. An overview of the results of the 10 regression analyses can be found in Fig 2. A table with the details concerning the overall significance of the models and details concerning the parameter estimates for the interactions and conditional effects is provided in S3 Appendix Table 2.

#### Susceptibility to normative influence predicts liking openness topics on Facebook

For the first regression analysis, we used the likelihood of liking low-openness topics as the response variable. Specifically, we used the mean of the likelihood rating of liking the 10 low-openness Facebook topics as the response variable (*M* = 19.08, *SD* = 24.12). SNI, openness, and their interaction were used as the predictor variables (mean centered). There was an interaction between SNI and openness (see Fig 2). A visual inspection of the interaction using the Johnson–Neyman technique (see S3 Appendix Figs 3–4) shows that higher levels of SNI (0.6 *SD* below or further above the mean) were associated with a stronger negative effect of openness on liking low-openness topics. Specifically, only for susceptible but not for unsusceptible OSN users, low (vs. high) openness increased the likelihood of liking low-openness topics. Further, there was a conditional positive effect of SNI. This means that the higher an “average openness user” scored on SNI, the more likely she or he has liked low-openness topics. There was also a conditional negative effect of openness. This means that the lower an “average susceptible user” scored on openness, the more likely she or he has liked low-openness topics (see Fig 2). Note that an ICC(2,*k*) of 0.95 shows that there was a good degree of agreement with which a participant indicated the likelihood of liking the 10 low-openness topics (*F*(543, 4887) = 21.0, *p* < 0.001, 95% CI [0.94, 0.96]). Hence, it seemed justified to build a mean score of the likelihood rating of these five Facebook topics.

For the second regression analysis, we used the mean of the likelihood rating of liking high-openness topics as the response variable (*M* = 24.68, *SD* = 19.08). SNI, openness, and their interaction were used as the predictor variables. There was an interaction between SNI and openness (see Fig 2). A visual inspection of the interaction using the Johnson–Neyman technique (see S3 Appendix Figs 5–6) shows that lower levels of SNI (0.05 *SD* above the mean or lower) were associated with a stronger positive effect of openness on liking high-openness topics. Specifically, only for unsusceptible but not for susceptible OSN users, high (vs. low) openness increased the likelihood of liking high-openness topics. Although there was a conditional positive effect of SNI, there was no conditional effect of openness (after Bonferroni correction; see Fig 2). Note that there was a good degree of agreement with which a participant indicated the likelihood of liking the 10 high-openness topics: ICC(2,*k*) = 0.93, *F*(543, 4887) = 16.0, *p* < 0.001, 95% CI [0.92, 0.94]).

#### Susceptibility to normative influence predicts liking conscientiousness topics on Facebook

For the third regression analysis, we used the mean of the likelihood rating of liking low-conscientiousness topics as a response variable (*M* = 20.57, *SD* = 23.36). SNI, conscientiousness, and their interaction were used as the predictor variables (mean centered). There was no interaction between SNI and conscientiousness (after Bonferroni correction). Although there was a conditional positive effect of SNI, there was no conditional effect of conscientiousness (see Fig 2). Note that there was a good degree of agreement with which a participant indicated the likelihood of liking the 10 low-conscientiousness topics: ICC(2,*k*) = 0.93, *F*(543, 4887) = 16.0, *p* < 0.001, 95% CI [0.92, 0.94]).

For the fourth regression analysis, we used the mean of the likelihood rating of liking high-conscientiousness topics as the response variable (*M* = 21.42, *SD* = 24.96). Again, SNI, conscientiousness, and their interaction were used as the predictor variables. There was no interaction between SNI and conscientiousness (see Fig 2). Although there was a conditional positive effect of SNI, there was no conditional effect of conscientiousness. Note that there was a good degree of agreement with which a participant indicated the likelihood of liking the 10 high-conscientiousness topics: ICC(2,*k*) = 0.95, *F*(543, 4887) = 4.9, *p* < 0.001, 95% CI [0.94, 0.96]).

#### Susceptibility to normative influence predicts liking extraversion topics on Facebook

For the fifth regression analysis, we used the mean of the likelihood rating of liking low-extraversion topics as the response variable (*M* = 30.28, *SD* = 25.36). SNI, extraversion, and their interaction were used as the predictor variables (mean centered). There was no interaction between SNI and extraversion (see Fig 2). Although there was a conditional positive effect of SNI, there was no conditional effect of extraversion. Note that there was a good degree of agreement with which a participant indicated the likelihood of liking the 10 low-conscientiousness topics: ICC(2,*k*) = 0.91, *F*(543, 4896) = 11.0, *p* < 0.001, 95% CI [0.90, 0.92]).

For the sixth regression analysis, we used the mean of the likelihood rating of liking high-extraversion topics as the response variable (*M* = 25.92, *SD* = 24.68). Again, SNI, extraversion, and their interaction were used as the predictor variables (mean centered). There was no interaction between SNI and extraversion (see Fig 2). There were, however, conditional positive effects of SNI and extraversion. Note that there was a good degree of agreement with which a participant indicated the likelihood of liking the 10 high-extraversion topics: ICC(2,*k*) = 0.95, *F*(543, 4896) = 14.0, *p* < 0.001, 95% CI [0.92, 0.94]).

#### Susceptibility to normative influence predicts liking agreeableness topics on Facebook

For the seventh regression analysis, we used the mean of the likelihood rating of liking low-agreeableness topics as the response variable (*M* = 22.41, *SD* = 22.81). SNI, agreeableness, and their interaction were used as the predictor variables (mean centered). There was an interaction between SNI and agreeableness (see Fig 2). A visual inspection of the interaction using the Johnson–Neyman technique (see S3 Appendix Figs 7–8) shows that higher levels of SNI (0.9 *SD* below or further above the mean) were associated with a stronger negative effect of agreeableness on liking low-agreeableness topics. Specifically, only for susceptible but not for unsusceptible OSN users, low (vs. high) agreeableness increased the likelihood of liking low-agreeableness topics. In addition, there was a conditional positive effect of SNI and a conditional negative effect of agreeableness (see Fig 2). Note that there was a good degree of agreement with which a participant indicated the likelihood of liking the 10 low-agreeableness topics: ICC(2,*k*) = 0.91, *F*(543, 4887) = 12.0, *p* < 0.001, 95% CI [0.90, 0.92]).

For the eighth regression analysis, we used the mean of the likelihood of liking high-agreeableness topics as the response variable (*M* = 19.35, *SD* = 24.18). Again, SNI, agreeableness, and their interaction were used as the predictor variables. There was an interaction between SNI and agreeableness (see Fig 2). A visual inspection of the interaction using the Johnson–Neyman technique (see S3 Appendix Figs 9–10) shows that higher levels of SNI (above the mean) were associated with a stronger negative effect of agreeableness on liking high-agreeableness topics. That is, for susceptible but not for unsusceptible OSN users, low (vs. high) agreeableness increased the likelihood of liking high-agreeableness topics. Although there was a conditional positive effect of SNI, there was no conditional effect of agreeableness (see Fig 2). Note that there was a good degree of agreement with which a participant indicated the likelihood of liking the 10 high-agreeableness topics: ICC(2,*k*) = 0.95, *F*(543, 4887) = 20.0, *p* < 0.001, 95% CI [0.94, 0.95]).

#### Susceptibility to normative influence predicts liking neuroticism topics on Facebook

For the ninth regression analysis, we used the mean of the likelihood rating of liking low-neuroticism topics as the response variable (*M* = 27.35, *SD* = 25.06). SNI, neuroticism, and their interaction were used as the predictor variables (mean centered). There was an interaction between SNI and neuroticism (see Fig 2). A visual inspection of the interaction using the Johnson–Neyman technique (see S3 Appendix Figs 11–12) shows that higher levels of SNI (0.25 *SD* below or further above the mean) were associated with a stronger positive effect of neuroticism when it came to liking low-neuroticism topics. Specifically, only for susceptible—not for unsusceptible OSN users—did high (vs. low) neuroticism increase the likelihood of liking low-neuroticism topics. Furthermore, there were conditional positive effects of SNI and neuroticism (see Fig 2). Note that there was a good degree of agreement with which a participant indicated the likelihood of liking the 10 low-openness topics: ICC(2,*k*) = 0.93, *F*(543, 4887) = 15.0, *p* < 0.001, 95% CI [0.92, 0.94]).

For the last regression analysis, we used the mean of the likelihood of liking high-neuroticism topics as the response variable (*M* = 22.37, *SD* = 23.98). Again, SNI, neuroticism, and their interaction were used as the predictor variables. There was an interaction between SNI and neuroticism (see Fig 2). A visual inspection of the interaction using the Johnson–Neyman technique (see S3 Appendix Figs 13–14) shows that higher levels of SNI were associated with a stronger positive effect of neuroticism on liking high-neuroticism topics. Specifically, only for susceptible—not for unsusceptible OSN users—did high (vs. low) neuroticism increase the likelihood of liking high-neuroticism topics. Further, there were conditional positive effects of SNI and neuroticism (see Fig 2). Note that there was a good degree of agreement with which the participants indicated the likelihood of liking the five high-neuroticism topics: ICC(2,*k*) = 0.93, *F*(543, 4887) = 16.0, *p* < 0.001, 95% CI [0.92, 0.94]).

## Discussion

The current research finds that the personality trait of SSI, namely the SNI facet—one’s tendency to conform to social norms to obtain approval from others—predicts behavior in OSNs such as Facebook. Specifically, we find that SSI correlates with diverse OSN behaviors. In fact, susceptible compared with unsusceptible users reported more often being affected by other OSN users, for example, to more often buy what other OSN users posted about, more often obtaining information about political issues from other OSN users, and more often liking Facebook topics because other OSN users had posted or commented on these topics (Studies 1a and 1b). The current research also indicates that the SNI facet of SSI exerts a stronger effect on OSN behavior when the topic of the behavior (e.g., fashion, politics, etc.) is perceived as important (Study 1b). Finally, the current research demonstrates that the SNI facet of SSI predicts to what extent OSN users hypothetically “like” online content (Studies 2 and 3). Exploring whether there is an interaction between SNI and the Big Five personality traits yielded mixed results. In fact, there is no consistent pattern regarding our idea that the Big Five personality traits would exert a stronger effect on OSN behavior for susceptible (vs. unsusceptible) OSN users.

The main finding of the current research is that OSN users’ SNI is a key factor for behavior in OSNs, hence determining the spread of change in OSN users’ attitudes, intentions, communication, and behaviors. In view of this, there are four promising avenues for future research. First, the understanding and prediction of social influence in OSNs might be improved by considering the personality trait of SSI. In fact, the current research indicates that SSI, in particular the SNI facet of SSI, is a better predictor for social influence in OSNs than personality traits (i.e., the Big Five personality traits) that have so far been the focus of research when it comes to understanding the spread of information and behavior in OSNs [21,24,41].

Second, it could be tested to what extent a psychometric measure of SSI relates to the metrics used for social network analyses as a way to identify susceptible users. Research using social network analyses could benefit from including a psychometric measure of SSI because this might allow for a more individual and psychological explanation of how information and behaviors spread in OSNs.

Third, the current research highlights OSN users’ vulnerability as being influenced by others in OSNs, finding that there is a group of individuals who are particularly vulnerable: people who scored high on the personality trait of SSI, that is, people who have a stable tendency to change attitudes, intentions, communication, and behaviors in response to others’ activities in OSNs. This finding is important for public authorities and researchers concerned with the harmful consequences of the dynamics of social influence and psychological targeting in OSNs. The current research can inform the implementation of political and legal measures against the harmful consequences of psychological targeting in OSNs. For example, future interventions to protect OSN users from the harmful consequences of psychological targeting should primarily address susceptible OSN users, making them aware of their susceptibility and providing them with the strategies to resist the influence of other OSN users.

Fourth, the present research underlines that we need to better understand how SSI interacts with other personality traits. Generally, the finding that SSI moderates the effect of some Big Five personality traits—openness, agreeableness, and neuroticisms—on hypothetical OSN behavior implies that combining some psychometric scales for the Big Five personality traits and SSI could improve the prediction of OSN behavior. For example, the interaction of SNI and openness on liking low-openness topics implies that OSN users who score low on openness and high on SNI are the most likely to like low-openness topics. Transferred to the real world, this means that susceptible compared with unsusceptible OSN users are particularly likely to comply with low-openness-related posts, such as those about Farmlandia (social farming game) or political campaigns with traditional values [24]. Thus, in the context of low-openness content, users who score low in openness and high on SNI seem to contribute to a comparably large extent to the social influence in OSNs. Yet regarding the interactions of SNI with openness, agreeableness, and neuroticism, we did not consistently find that higher SNI leads to a stronger effect of the Big Five personality traits on the likelihood of liking Facebook topics that are indicative of the respective Big Five personality traits. For example, we expected that susceptible compared with unsusceptible OSN users would be more likely to like high-agreeableness topics such as “Christianity” when they scored high (vs. low) on agreeableness. Yet we found that susceptible OSN users were more likely to like such high-agreeableness topics when they scored low (vs. high) on agreeableness. Clearly, this is not intuitive, and it should be noted that this originates from failing to replicate the main effect of agreeableness [24,41]. In fact, our data could not support the basic assumption that higher agreeableness would be associated with a higher likelihood of liking high-agreeableness topics. Given that we did not find this “main effect” of agreeableness, it is difficult to make concluding remarks on the question of whether SNI moderates the effect of the Big Five personality traits on OSN behavior.

Certainly, the current research has limitations. It can be argued that this research lacks a stringent definition and specific operationalization of social influence in OSNs. When testing whether SSI predicts OSN behavior, we considered the OSN behaviors of various degrees of social influence. We considered OSN behaviors such as paying attention to certain information because of the influence of other OSN users (e.g., reading other OSN users’ posts on political content), as well as actually showing certain behaviors because of the influence of other OSN users (e.g., purchasing products that the individual has become aware of through other OSN users’ posts). Clearly, one could argue that paying attention to certain information because of other OSN users’ influence does not necessarily mean that one also changes one’s attitudes and behaviors [42]. Referring to research using social network analyses, which often focuses on how information spreads in OSNs, we explicitly consider the mere spread of information because of the influence of other OSN users as “social influence.” A further criticism is that Studies 2 and 3 are based on Facebook topics that might be somewhat outdated. The Facebook topics (e.g., My calendar 2010) that we used originated from the work of Kosinski, Stillwell, and Graepel, which was published in 2013. Likewise, the psychometric scales that we used to capture SSI are outdated, base on “offline” social influence principles that partly do not apply to the online environment and capture SSI specific to an unrelated context (i.e., consumption), which is likely to result in a suboptimal prediction of OSN users’ susceptibility. Another important limitation is that the present research is correlational and relies on hypothetical self-reported behavior. It would be interesting to run (field) experiments and test whether susceptible (vs. unsusceptible) OSN users are really more likely to show behaviors that lead to effective online social influence in OSNs. Additionally, it should be noted that the sample size of Studies 1a and 1b are rather small, and we cannot rule out that some people in Studies 2 and 3 indicated to “very likely” like a topic just because they had already liked the topic. Future studies that test the influence of SSI on Facebook liking should differ between the possibility that a topic has already been liked and whether someone likes a topic because others have liked it. A critical aspect is also that the incentives in Studies 1a and 1b differ from the incentives in Studies 2 and 3 and that data were collected online and via MTurk, which may have some drawbacks, such as that the samples may not be representative of the general population and that the participants filled in the survey in a less controlled setting. Despite these limitations, our findings provide valuable information on the relationship between SSI and OSN behavior, indicating that there is a significant relationship between these two constructs. Exploring this relationship further will require more research.

## Supporting information

S1 Appendix Additional details of Studies 1a and 1b. (DOCX)

S2 Appendix Additional details of Study 2. (DOCX)

S3 Appendix Additional details of Study 3. (DOCX)
