# Supplementary material for: Susceptibility to social influence predicts behavior on Facebook
Source: PLoS One. 2020 Mar 3;15(3):e0229337. doi: 10.1371/journal.pone.0229337 (PMC7053739; doi:10.1371/journal.pone.0229337)
Supplement: S1 Appendix — (DOCX) [file pone.0229337.s001.docx]

**SI Appendix 1: Additional details of Studies 1a and 1b**

Table S1.1: Online social network (OSN) behaviors

| **OSN behavior related to the topic of…** | **Question** |
| --- | --- |
| Fashion | I have been inspired by the fashion and style of other Facebook users (e.g., photos, posts). |
| Politics | I have read other Facebook users' posts on political content. |
| Products/brands | I have noticed certain products or brands in posts from other Facebook users. |
| News | Through posts from other Facebook users I have learned about news (e.g., politics, sports). |
| Food | I have looked at posts from other Facebook users on food trends (e.g., recipes). |
| Music | I listened to music I discovered on Facebook. |
| Forwarding posts | I have forwarded/shared posts from other Facebook users. |
| Liking posts | I have liked or commented on posts by other Facebook users. |
| Events | I have participated in events that I have become aware of through Facebook users. |
| Impact on offline life | I have talked about Facebook content outside of social media (i.e., personally). |
| Purchase of products/brands | I have purchased products or brands that I have become aware of through posts from other Facebook users. |
| Charity | I have supported charity actions on Facebook (e.g., the ALS [Amyotrophic Lateral Sclerosis] Ice Bucket Challenge). |
| Traveling | I have visited places (e.g., city, country, museum, park) that I have become aware of through posts from other Facebook users. |
| Joining pages | I have joined Facebook pages recommended to me by friends. |
| Perceived social influence | I have been influenced by Facebook content from other Facebook users. |

15 OSN behaviors operationalized as behaviors in Facebook that are an expression of engaging in social influence. The behaviors are defined for different topics (e.g., politics, brands, music, traveling, food; see left column). The participants indicated for all OSN behaviors how frequently they engage in these behaviors (from 1 = *never* to 5 = *always*).

Table S1.2: Description of the susceptibility-to-social-influence scales (SSI) scales (Studies 1a and 1b)

| **Facets of SSI (SSI scales)** | **Description** |
| --- | --- |
| Susceptibility to normative influence (1) | One’s tendency to comply to social norms regarding products, services, and brands; subscale of consumer susceptibility to interpersonal influence scale. |
| Susceptibility to informative influence (1) | One’s tendency to learn about products, services, and brands by seeking information from others; subscale of consumer susceptibility to interpersonal influence scale. |
| Attention to social comparison information (2) | One’s tendency to pay attention to and be concerned about reactions to one’s behavior. |
| Information seeking (3) | One’s tendency to seeks information about brands and fashion from others; subscale of interpersonal communication and influence scale. |
| Public self-consciousness (4) | One’s tendency to be aware of the impression one makes on others and for the extent to which one is concerned about this. |
| Need for consistency (5) | One’s desire to be consistent within one's own responses and appear consistent to others. |
| Lack of skepticism (6) | One’s tendency toward disbelieving advertising claims. |
| Need for uniqueness (7) | One’s desire to feel differentiated from other consumers, namely, the need for avoidance of similarity; subscale of consumer need for uniqueness scale. |

Overview and description of the psychometric scales that we have used to capture the different facets of SSI.

To identify the SSI scales, we reviewed the compilation books *Handbook of Marketing Scales* (8) and *Measures of Personality and Social Psychological Constructs* (9). We also conducted an online research using Google Scholar (search terms: “online social network” + “social influence” and “online social network” + “psychometric scale”). Here, we used the first 10 pages of the Google Scholar search results. In doing so, we considered those psychometric scales that we perceived as qualified to capture different facets of SSI, that is, the tendency to change attitudes, intentions, communication, and behavior because of others in the OSN. All items of the described scales are provided in Table S1.3.

Table S1.3: SSI scales (Studies 1a and 1b)

**Susceptibility-to-normative-influence scale**

If I want to be like someone, I often try to buy the same brands that they buy.
It is important that others like the products and brands I buy.
I rarely purchase the latest fashion styles until I am sure my friends approve of them.
I often identify with other people by purchasing the same products and brands they purchase.
When buying products, I generally purchase those brands that I think others will approve of.
I like to know what brands and products make good impressions on others.
If other people can see me using a product, I often purchase the brand they expect me to buy.
I achieve a sense of belonging by purchasing the same products and brands that others purchase.

**Susceptibility-to-informative-influence scale**

I often consult other people to help choose the best alternative available from a product class.
To make sure I buy the right product or brand, I often observe what others are buying and using.
If I have little experience with a product, I often ask my friends about the product.
I frequently gather information from friends or family about a product before I buy.

**Attention-to-social-comparison-information scale**

It is my feeling that if everyone else in a group is behaving in a certain manner, this must be the proper way to behave.
I actively avoid wearing clothes that are not in style.
At parties I usually try to behave in a manner that makes me fit in.
When I am uncertain how to act in a social situation, I look to the behavior of others for clues.
I try to pay attention to the reactions of others to my behavior in order to avoid being out of place.
I find that I tend to pick up slang expressions from others and use them as a part of my own vocabulary.
I tend to pay attention to what others are wearing.
The slightest look of disapproval in the eyes of a person with whom I am interacting is enough to make me change my approach.
It's important to me to fit into the group I'm with.
My behavior often depends on how I feel others wish me to behave.
If I am the least bit uncertain as to how to act in a social situation, I look to the behavior of others for clues.
I usually keep up with clothing style changes by watching what others wear.
When in a social situation, I tend not to follow the crowd, but instead to behave in a manner that suits my particular mood at the time.*

**Information-seeking scale**

I often seek out the advice of my friends regarding which clothes I buy.

I spend a lot of time talking with my friends about clothing fashions.

My friends or neighbors usually give me good advice on what brands of clothes to buy.

**Public-self-consciousness scale**

I’m concerned about my style of doing things.
I care a lot about how I present myself to others.
I’m self-conscious about the way I look.
I usually worry about making a good impression.
Before I leave my house, I check how I look.
I’m concerned about what other people think of me.
I’m usually aware of my appearance.

**Need-for-consistency scale**

It is important to me that those who know me can predict what I will do.
I want to be described by others as a stable, predictable person.
The appearance of consistency is an important part of the image I present to the world.
An important requirement for any friend of mine is personal consistency.
I typically prefer to do things the same way.
I want my close friends to be predictable.
It is important to me that others view me as a stable person.
I make an effort to appear consistent to others.
It doesn't bother me much if my actions are inconsistent.*

**Lack-of-skepticism scale**

We can depend on getting the truth in most advertising.
Advertising's aim is to inform the consumer.
I believe advertising is informative.
Advertising is generally truthful.
Advertising is a reliable source of information about the quality and performance of products.
Advertising is truth well told.
In general, advertising presents a true picture of the product being advertised.
I feel I've been accurately informed after viewing most advertisements.
Most advertising provides consumers with essential information.

**Need-for-uniqueness scale**

When products or brands I like become extremely popular, I lose interest in them.

I avoid products or brands that have already been accepted and purchased by the average consumer.

When a product I own becomes popular among the general population, I begin using it less.

I often try to avoid products or brands that I know are bought by the general population.

As a rule, I dislike products or brands that are customarily purchased by everyone.

I give up wearing fashions I've purchased once they become popular among the general public.

The more commonplace a product or brand is among the general population, the less interested I am in buying it.

Products don't seem to hold much value for me when they are purchased regularly by everyone.

When a style of clothing I own becomes too commonplace, I usually quit wearing it.

For all SSI scales, a 5-point Likert scale was used (from disapproval to approval). The items that are reverse scored are marked with an asterisk (*).

Fig. S1.1: Heat map with all the correlations between SSI scales and OSN behavior in Studies 1a and 1b


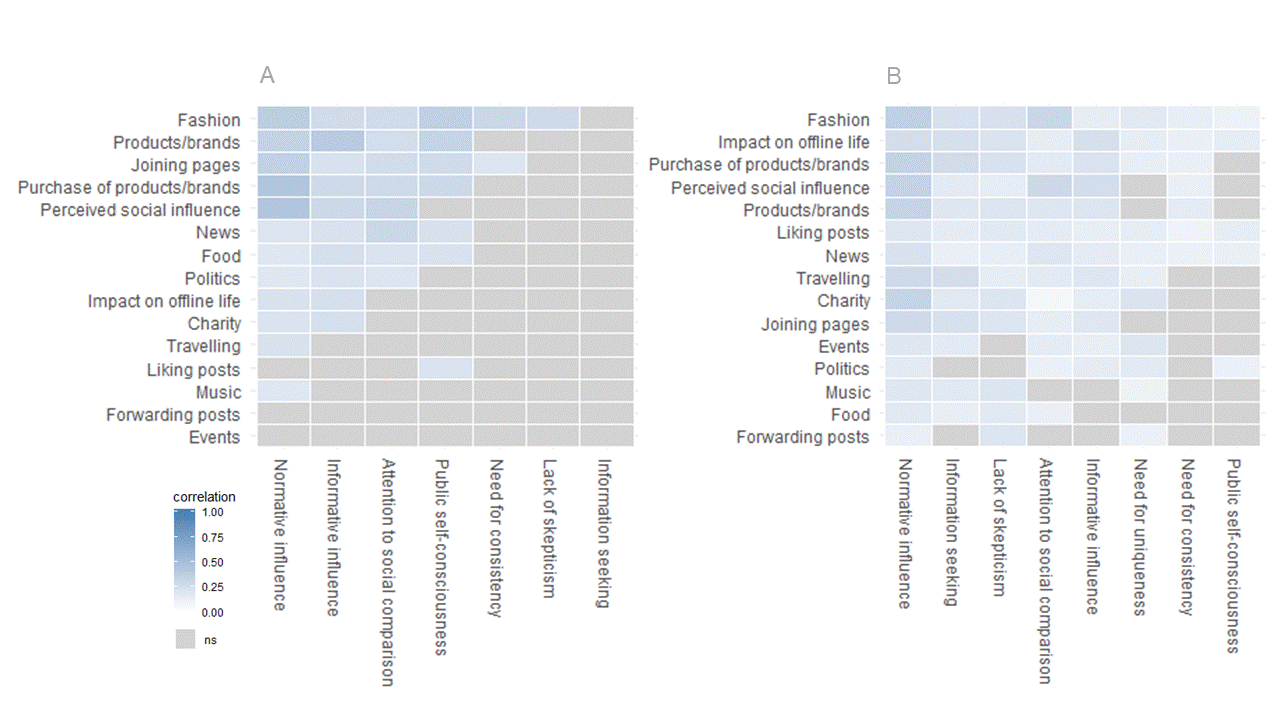


Heat map of correlations of SSI scales and 15 OSN behaviors. Fig. S1.1A depicts the results of Study 1a (7 SSI scales). Fig. S.1.1B depicts the results of Study 1b (8 SSI scales). The correlations are coded from 0 (white) to 1 (dark blue). Non-significant correlations are depicted in gray (ns). Note that in Study 1b, we used one more psychometric scale than in Study 1a (i.e., the need-for-uniqueness scale).

Table S1.4: Correlations between SSI scales and behavior in Online Social Networks (Study 1a)

| **OSN Behaviors** | **Normative**  **Influence^a^** | **Informative influence^b^** | **Attention to social comparison^c^** | **Public self-consciousness^d^** | **Need for consistency^e^** | **Lack of skepticism^f^** | **Information seeking^g^** |
| --- | --- | --- | --- | --- | --- | --- | --- |
| Fashion | **0.38*****  **[0.10, 0.63]** | **0.27****  **[0.04, 0.49]** | **0.27****  **[0.05, 0.48]** | **0.36*****  **[0.20, 0.50]** | **0.30*****  **[0.10, 0.49]** | **0.28****  **[0.05, 0.51]** | 0.13  [-0.06, 0.32] |
| Politics | **0.18***  **[0.00, 0.34]** | **0.21***  **[0.01, 0.38]** | 0.20*  [0.01, 0.36] | 0.12  [-0.06, 0.30] | 0.12  [-0.07, 0.30] | -0.06  [-0.24, 0.13] | 0.13  [-0.06, 0.29] |
| Products/brands | **0.34*****  **[0.13, 0.52]** | **0.40*****  **[0.20, 0.55]** | **0.25****  **[0.08, 0.41]** | **0.33*****  **[0.16, 0.46]** | 0.03  [-0.14, 0.21] | 0.11  [-0.10, 0.31] | 0.14  [-0.06, 0.32] |
| News | **0.20***  **[0.05, 0.41]** | **0.22***  **[0.01, 0.40]** | **0.30****  **[0.14, 0.47]** | **0.23***  **[0.06, 0.41]** | 0.11  [-0.07, 0.28] | 0.02  [-0.18, 0.19] | 0.06  [-0.16, 0.24] |
| Food | **0.18***  **[0.02, 0.39]** | **0.24****  **[0.06, 0.40]** | **0.21***  **[0.03, 0.40]** | **0.22***  **[0.04, 0.38]** | 0.09  [-0.10, 0.26] | 0.06  [-0.10, 0.21] | 0.13  [-0.05, 0.30] |
| Music | **0.18***  **[-0.02, 0.36]** | 0.14  [-0.05, 0.31] | 0.00  [-0.19, 0.19] | 0.16  [-0.02, 0.33] | 0.12  [-0.04, 0.26] | 0.13  [-0.07, 0.30] | -0.06  [-0.23, 0.12] |
| Forwarding posts | 0.17  [-0.04, 0.37] | 0.16  [-0.04, 0.33] | 0.08  [-0.12, 0.25] | 0.17  [0.00, 0.32] | 0.09  [-0.08, 0.26] | 0.02  [-0.20, 0.25] | 0.12  [-0.06, 0.29] |
| Liking posts | 0.13  [-0.05, 0.32] | 0.10  [-0.10, 0.29] | 0.07  [-0.11, 0.26] | **0.21***  **[0.03, 0.38]** | 0.13  [-0.08, 0.32] | 0.13  [-0.07, 0.30] | -0.03  [-0.23, 0.15] |
| Event | 0.08  [-0.13, 0.27] | 0.05  [-0.18, 0.23] | 0.05  [-0.11, 0.20] | -0.02  [-0.19, 0.15] | 0.08  [-0.07, 0.26] | -0.04  [-0.24, 0.17] | 0.08  [-0.10, 0.24] |
| Impact on offline life | **0.22***  **[0.06, 0.36]** | **.0.24****  **[0.07, 0.39]** | 0.02  [-0.15, 0.18] | **0.17***  **[0.02, 0.35]** | 0.04  [-0.14, 0.22] | 0.03  [-0.17, 0.23] | 0.10  [-0.09, 0.28] |
| Purchase of products/brands | **0.44*****  **[0.12, 0.70]** | **0.28*****  **[0.02, 0.53]** | **0.28****  **[0.07, 0.50]** | **0.29****  **[0.09, 0.46]** | 0.10  [-0.11, 0.31] | 0.07  [-0.16, 0.35] | 0.10  [-0.10, 0.32] |
| Charity | **0.21***  **[-0.03, 0.57]** | **0.24****  **[0.09, 0.47]** | 0.11  [-0.08, 0.35] | -0.08  [-0.27, 0.10] | -0.04  [-0.25, 0.23] | 0.03  [-0.21, 0.41] | 0.07  [-0.11, 0.28] |
| Traveling | **0.22***  **[0.01, 0.44]** | 0.16  [-0.09, 0.38] | 0.14  [-0.04, 0.30] | 0.05  [-0.12, 0.21] | 0.09  [-0.08, 0.27] | 0.01  [-0.22, 0.26] | 0.03  [-0.15, 0.21] |
| Joining pages | **0.35*****  **[0.11, 0.59]** | **0.22***  **[-0.01, 0.44]** | **0.26****  **[0.06, 0.45]** | **0.27****  **[0.14, 0.41]** | 0.20*  [0.02, 0.37] | 0.17  [-0.04, 0.40] | 0.13  [-0.06, 0.32] |
| Perceived social influence | **0.44*****  **[0.20, 0.66]** | **0.29*****  **[0.06, 0.51]** | **0.32*****  **[0.12, 0.51]** | 0.15  [-0.03, 0.32] | 0.14  [-0.07, 0.34] | 0.12  [-0.09, 0.43] | 0.16  [-0.03, 0.35] |

Pearson correlation coefficients with 95% bootstrap BCa CIs for susceptibility-to-online-social-influence scales and OSN behaviors. **p* < 0.05. ***p* < 0.01. ****p* < 0.001.

^a^ Susceptibility-to-normative-influence scale (α = 0.89), ^b^ Susceptibility-to-informative-influence scale (α = 0.80), ^c^ Attention-to-social-comparison-information scale (α = 0.88), ^d^ Public-self-consciousness scale (α = 0.82), ^e^ Preference-for-consistency scale (α = 0.83), ^f^ Lack-of-skepticism scale (α = 0.91), ^g^ Information-seeking scale (α = 0.71).

Note that the bold correlations are also significant after using the Benjamini–Hochberg procedure for every susceptibility-to-online-social-influence scale with a 10% false discovery rate.

Table S1.5: Correlations between the SSI scales and behavior in online social networks (Study 1b)

| **OSN Behaviors** | **Normative influence^a^** | **Information seeking^b^** | **Lack of skepticism^c^** | **Attention to social comparison^d^** | **Informative influence^e^** | **Need for uniqueness^f^** | **Need for consistency^g^** | **Public self-consciousness^h^** |
| --- | --- | --- | --- | --- | --- | --- | --- | --- |
| Fashion | **0.36*****  **[0.25, 0.48]** | **0.23*****  **[0.12, 0.34]** | **0.23*****  **[0.11, 0.34]** | **0.31*****  **[0.22, 0.40]** | **0.14****  **[0.03, 0.25]** | **0.17*****  **[0.05, 0.26]** | **0.13***  **[0.03, 0.23]** | 0.10*  [0.00, 0.22] |
| Politics | **0.16*****  **[0.06, 0.28]** | 0.08  [-0.03, 0.18] | -0.03  [-0.13, 0.08] | **0.11***  **[0.01, 0.21]** | **0.15****  **[0.04, 0.26]** | **0.16*****  **[0.06, 0.26]** | 0.07  [-0.04, 0.18] | 0.11*  [0.00, 0.22] |
| Products/brands | **0.33*****  **[0.22, 0.43]** | **0.18*****  **[0.07, 0.28]** | **0.20*****  **[0.07, 0.30]** | **0.19*****  **[0.09, 0.30]** | **0.20*****  **[0.13, 0.34]** | 0.09  [-0.03, 0.19] | **0.15****  **[0.04, 0.25]** | 0.13  [-0.02, 0.19] |
| News | **0.22*****  **[0.11, 0.33]** | **0.12****  **[0.02, 0.22]** | **0.13***  **[0.03, 0.24]** | **0.19*****  **[0.10, 0.28]** | **0.15****  **[0.04, 0.26]** | **0.12***  **[0.03, 0.22]** | **0.11***  **[0.00, 0.22]** | 0.12*  [0.00, 0.22] |
| Food | **0.17*****  **[0.06, 0.28]** | **0.13****  **[0.01, 0.24]** | **0.17*****  **[0.07, 0.27]** | **0.12***  **[0.02, 0.21]** | 0.10  [0.00, 0.20] | 0.01  [-0.10, 0.12] | 0.05  [-0.05, 0.15] | 0.02  [-0.09, 0.12] |
| Music | **0.18*****  **[0.06, 0.31]** | **0.17*****  **[0.06, 0.27]** | **0.20*****  **[0.10, 0.31]** | 0.07  [-0.02, 0.18] | 0.05  [0.-0.05, 0.16] | **0.10***  **[-0.01, 0.20]** | 0.01  [-0.10, 0.12] | 0.05  [-0.15, 0.05] |
| Forwarding posts | **0.12***  **[0.00, 0.25]** | 0.06  [-0.05, 0.17] | **0.20*****  **[0.10, 0.30]** | 0.00  [-0.10, 0.10] | 0.06  [-0.04, 0.16] | **0.11***  **[0.01, 0.21]** | 0.05  [-0.05, 0.15] | -0.03  [-0.12, 0.08] |
| Liking posts | **0.19****  **[0.03, 0.25]** | **0.15****  **[0.08, 0.30]** | **0.17*****  **[0.06, 0.27]** | **0.15****  **[0.05, 0.24]** | **0.13****  **[0.03, 0.22]** | **0.14****  **[0.04, 0.24]** | 0.09*  [-0.02, 0.20] | 0.14**  [0.03, 0.24] |
| Event | **0.18*****  **[0.08, 0.29]** | **0.16*****  **[0.06, 0.26]** | **0.09**  **[-0.02, 0.19]** | **0.15****  **[0.05, 0.24]** | **0.13****  **[0.03, 0.23]** | **0.20*****  **[0.10, 0.29]** | -0.02  [-0.12, 0.07] | 0.03  [-0.07, 0.13] |
| Impact on offline life | **0.25*****  **[0.14, 0.35]** | **0.24*****  **[0.14, 0.35]** | **0.21*****  **[0.11, 0.31]** | **0.14****  **[0.05, 0.23]** | **0.24*****  **[0.14, 0.33]** | **0.14****  **[0.04, 0.24]** | **0.12***  **[0.02, 0.22]** | 0.14*  [0.00, 0.21] |
| Purchase of products/brands | **0.34*****  **[0.22, 0.43]** | **0.26*****  **[0.15, 0.37]** | **0.22*****  **[0.12, 0.32]** | **0.16****  **[0.07, 0.25]** | **0.21*****  **[0.17, 0.40]** | **0.13****  **[0.03, 0.23]** | **0.12***  **[0.02, 0.23]** | 0.02  [-0.09, 0.11] |
| Charity | **0.33*****  **[0.21, 0.43]** | **0.17*****  **[0.05, 0.30]** | **0.20*****  **[0.09, 0.29]** | 0.06**  [-0.06, 0.15] | **0.14****  **[0.05, 0.22]** | **0.21***  **[0.02, 0.22]** | 0.15  [0.00, 0.16] | -0.08  [-0.17, 0.01] |
| Traveling | **0.28*****  **[0.15, 0.41]** | **0.25*****  **[0.15, 0.35]** | **0.14****  **[0.04, 0.26]** | **0.16****  **[0.05, 0.27]** | **0.19*****  **[0.08, 0.30]** | **0.13***  **[0.02, 0.23]** | 0.03  [-0.08, 0.13] | 0.01  [-0.09, 0.11] |
| Joining pages | **0.28*****  **[0.16, 0.38]** | **0.23*****  **[0.17, 0.41]** | **0.20*****  **[0.08, 0.30]** | **0.14****  **[0.04, 0.24]** | **0.18*****  **[0.08, 0.27]** | 0.08  [-0.02, 0.18] | 0.05  [-0.05, 0.15] | 0.01  [-0.09, 0.11] |
| Perceived social influence | **0.34*****  **[0.22, 0.47]** | **0.16****  **[0.12, 0.34]** | **0.14****  **[0.09, 0.41]** | **0.29*****  **[0.18, 0.40]** | **0.25*****  **[0.15, 0.35]** | 0.06  [-0.04, 0.16] | **0.12***  **[0.02, 0.22]** | 0.08  [-0.03, 0.17] |

Pearson correlation coefficients with 95% bootstrap BCa CIs for susceptibility-to-online-social-influence scales and 15 OSN behaviors. **p* < .05. ***p* < .01. ****p* < .001.

^a^ Susceptibility-to-normative-influence scale (α = 0.89), ^b^ Information-seeking scale (α = 0.75), ^c^ Lack-of-skepticism scale (α = 0.88), ^d^ Attention-to-social-comparison-information scale (α = 0.86), ^e^ Susceptibility-to-informative-influence scale (α = 0.78), ^f^ Need-for-uniqueness scale (α = 0.84). ^g^ Preference-for-consistency scale (α = 0.82),

^h^ Public-self-consciousness scale (α = 0.72).

Note that the bold correlations are also significant after using the Benjamini–Hochberg procedure for every susceptibility-to-online-social-influence scale with a 10% false discovery rate.

S1.6: Model and computational details

model <- lmer(OSNbehavior ~ CSII_Norm_M.gmc * Importance.gmc + (1 | ID) + (1 | BehaviorTopic), data = InflData_long)

summary(model)

We used the lmer function of the lme4 package of R (Version 1.1–21; 33) to fit the model. Note that we grand mean centered (gmc) the two fixed factors, that is, CSII_Norm_M.gmc = CSII_Norm_M-mean(CSII_Norm_M)

Importance.gmc = Importance-mean(Importance)

ID = participant identifier, BehaviorTopic = identifier for the nine different OSN behaviors.
