# Supplementary material for: Susceptibility to social influence predicts behavior on Facebook
Source: PLoS One. 2020 Mar 3;15(3):e0229337. doi: 10.1371/journal.pone.0229337 (PMC7053739; doi:10.1371/journal.pone.0229337)
Supplement: S2 Appendix — (DOCX) [file pone.0229337.s002.docx]

**SI Appendix 2: Additional details of Study 2**

Table S2.1: Facebook topics that are indicative for extraversion and openness

|  | Extraversion | Openness |
| --- | --- | --- |
| High | Beerpong  Dancing  Socializing  Cheerleading  Theatre | Oscar Wilde  Leonardo Da Vinci  American Gods  Plato  Leonard Cohen |
| Low | Programming  Anime  Role playing games  Video games  Minecraft | NASCAR  I don’t read  ESPN2  The Bachelor  Teen Mom |

These four sets of Facebook topics are indicative for low/high extraversion and low/high openness. Note that the topics stem from previous research (Kosinski et al., 2015) identifying Facebook likes that are indicative of the Big Five personality traits.

Note that for each of the four categories (i.e., low extraversion, high extraversion, low openness, and high openness), we choose five of the 10 Facebook likes provided in the supplementary material in Kosinski et al., 2015.

Table S2.2: Scales for the Big Five personality traits (Studies 2 and 3)

**Openness**

Have a vivid imagination.

Carry the conversation to a higher level.

Believe in the importance of art.

Tend to vote for liberal political candidates.

Enjoy hearing new ideas.

Am not interested in abstract ideas.*

Do not like art.*

Avoid philosophical discussions.*

Do not enjoy going to art museums.*

Tend to vote for conservative political candidates.*

**Conscientiousness**

Am always prepared.

Pay attention to details.

Get chores done right away.

Make plans and stick to them.

Carry out my plans.

Waste my time.*

Shirk my duties.*

Find it difficult to get down to work.*

Do just enough work to get by.*

Don't see things through.*

**Extraversion**

Feel comfortable around people.

Am the life of the party.

Am skilled in handling social situations.

Make friends easily.

Know how to captivate people.

Don't talk a lot.*

Have little to say.*

Keep in the background.*

Don't like to draw attention to myself.*

Would describe my experiences as somewhat dull.*

**Agreeableness**

Make people feel at ease.

Have a good word for everyone.

Believe that others have good intentions.

Respect others.

Accept people as they are.

Insult people.*

Have a sharp tongue.*

Cut others to pieces.*

Suspect hidden motives in others.*

Get back at others.*

**Neuroticism**

Often feel blue.

Have frequent mood swings.

Panic easily.

Dislike myself.

Am often down in the dumps.

Seldom feel blue.*

Am not easily bothered by things.*

Rarely get irritated.*

Feel comfortable with myself.*

Am very pleased with myself.*

We used the short International Personality Item Pool questionnaire (Costa & McCrae, 1992) to capture the Big Five personality traits. For all susceptibility-to-social-influence scales, a 5-point Likert scale was used (from disapproval to approval). Items that are reverse scored are marked with an asterisk (*).

Table S2.3: Parameter estimates for the regression analyses with SNI and openness/extraversion on OSN behaviors

|  | *Β* | SE | 95% CI for *B* | *t* | *p* |
| --- | --- | --- | --- | --- | --- |
| Response variable: likelihood of liking low-openness topics | | | | | |
| (Intercept) | 9.46 | 0.86 | [7.77, 11.16] | 10.98 | < 0.001 |
| Openness | -2.02 | 1.23 | [-4.44, 0.39] | -1.65 | 0.100 |
| SNI | 6.59 | 0.97 | [4.68, 8.51] | 6.77 | **< 0.001** |
| Openness*SNI | -4.14 | 1.25 | [-6.60, -1.68] | -3.31 | **0.001** |
| Response variable: likelihood of liking high-openness topics | | | | | |
| (Intercept) | 17.45 | 1.18 | [15.13, 19.76] | 14.81 | < 0.001 |
| Openness | 7.32 | 1.67 | [4.03, 10.61] | 4.38 | **< 0.001** |
| SNI | 6.46 | 1.33 | [3.84, 9.07] | 4.87 | **< 0.001** |
| Openness*SNI | -5.75 | 1.70 | [-9.10, -2.40] | -3.38 | **< 0.001** |
| Response variable: likelihood of liking low-extraversion topics | | | | | |
| (Intercept) | 28.58 | 1.35 | [25.91, 31.24] | 21.12 | < 0.001 |
| Extraversion | -0.66 | 1.78 | [-4.54, 2.47] | -0.58 | 0.561 |
| SNI | 5.14 | 1.55 | [1.74, 7.85] | 3.09 | **< 0.01** |
| Extraversion*SNI | 1.79 | 1.85 | [-2.31, 4.99] | 0.72 | 0.470 |
| Response variable: likelihood of liking high-extraversion topics | | | | | |
| (Intercept) | 18.72 | 1.06 | [16.64, 20.81] | 17.71 | < 0.001 |
| Extraversion | 4.41 | 1.39 | [1.68, 7.16] | 3.17 | **< 0.01** |
| SNI | 8.29 | 1.21 | [5.90, 10.68] | 6.84 | **< 0.001** |
| Extraversion*SNI | 0.00 | 1.45 | [-2.85, 2.85] | 0.00 | 0.999 |

The parameter estimates for the conditional effects and interactions of SNI and extraversion/openness on Facebook liking behavior.

Overall significance of the model for low-openness topics: *F*(3, 290) = 23.66, *p* < 0.001, adjusted *R^2^* = 0.19 (4 missingness); high-openness topics: *F*(3, 290) = 14.50, *p* < 0.001, adjusted *R^2^* = 0.12 (4 missingness); low-extraversion topics: *F*(3, 292) = 3.88, *p* < 0.01, adjusted *R^2^* = 0.03 (2 missingness); high-extraversion topics: *F*(3, 292) = 21.12, *p* < 0.001 adjusted *R^2^* = 0.17 (2 missingness). Note that all the variance inflation factors (VIFs) are < 2 (hence there is no problem with multicollinearity) and that the bold *p* values are also significant after Bonferroni correction (0.05 level).

Figure S2.4: SNI as moderator of the effect of openness on liking low-openness topics^[[1]](#footnote-1)^


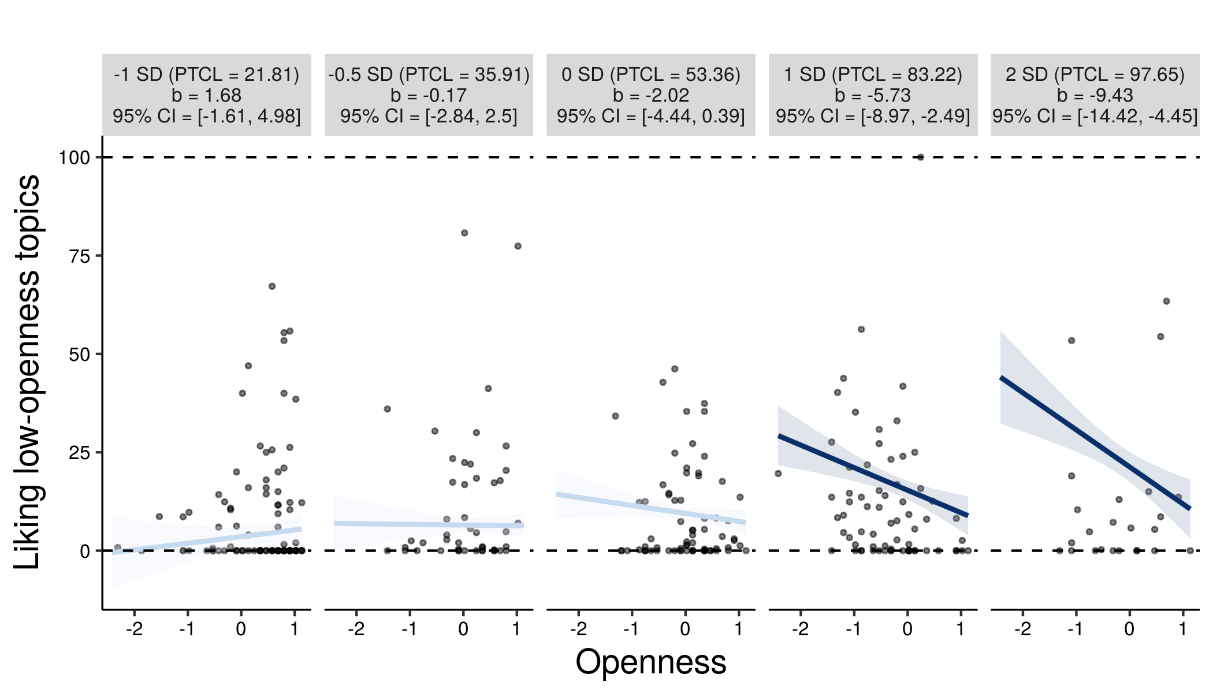


Simple slopes plot of the interaction of SNI and openness. The x axes represent openness (range of observed data for the focal predictor). Simple slopes are depicted for the levels of SNI (moderator) 1 SD and 0.5 SD below the mean, as well as 1 SD and 2 SD above the mean. Note that we chose different SD deviations from the mean to best display the observed data. For all the slopes, we depict the 95% confidence region (shaded area), observed data (data points), and the maximum and minimum values of the response variable (dashed lines). CI = confidence interval, PTCL = percentile.

Figure S2.5: Johnson-Neyman plot (liking low-openness topics)


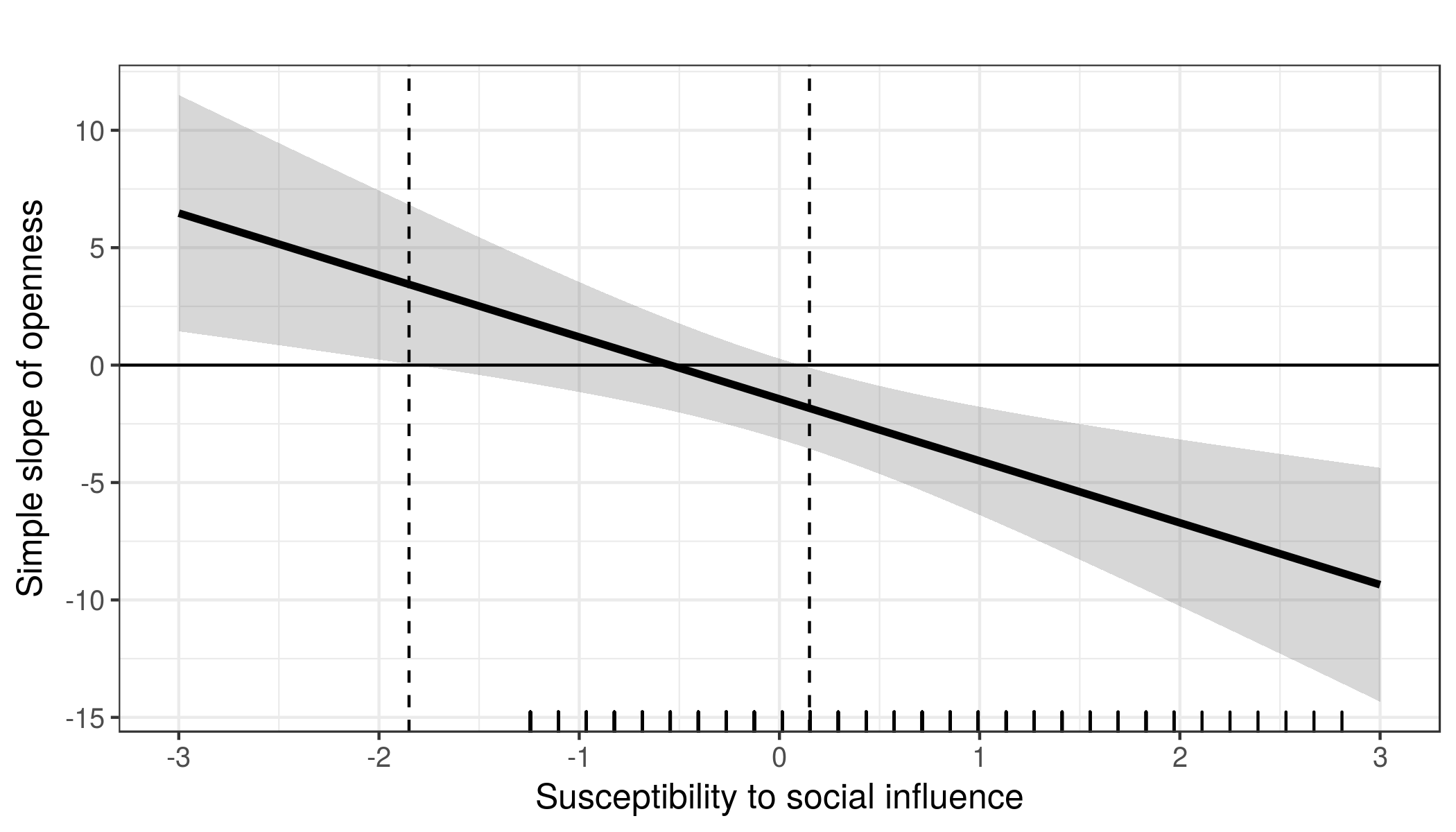


Visualization of the simple slope of openness on liking low-openness topics across the full range of SNI. The simple slope of openness on liking low-openness topics is significant outside the interval of [-1.62, 0.10] and negative when SNI is 0.15 SD or further above the mean (43% of the observations are within this region). Although the Johnson-Neyman plot reveals that the simple slope of openness is also significantly positive when SNI is 1.9 SD or further below the mean, there are 0% of the observed data in this region. The dashed vertical lines indicate the levels of SNI at which openness becomes significantly associated with liking low-openness topics.

Figure S2.6: SNI as a moderator of the effect of openness on liking high-openness topics


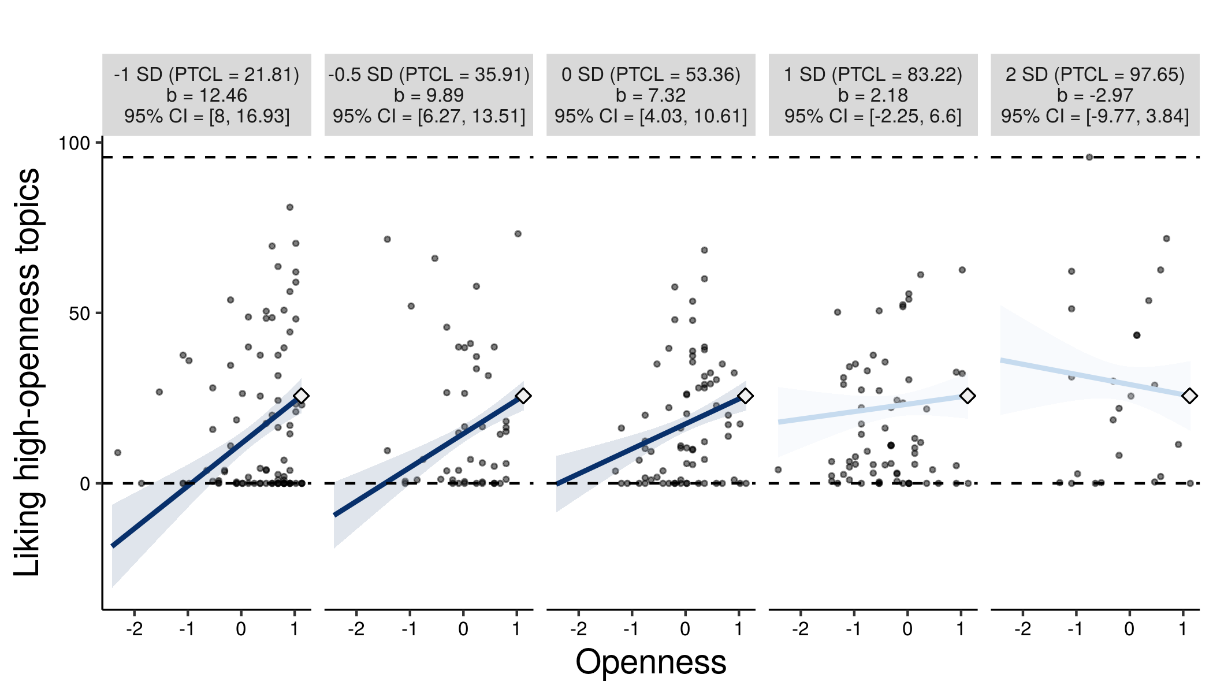


Simple slopes plot of the interaction of SNI and openness. The x axes represent openness (range of observed data for the focal predictor). Simple slopes are depicted for levels of SNI (moderator) 1 SD and 0.5 SD below the mean, as well as 1 SD and 2 SD above the mean. Note that we chose different SD deviations from the mean to best display the observed data. For all the slopes, we depict the 95% confidence region (shaded area), observed data (data points), the maximum and minimum values of the response variable (dashed lines), and the crossover point (diamond). CI = confidence interval, PTCL = percentile.

Figure S2.7: Johnson-Neyman plot (liking high-openness topics)


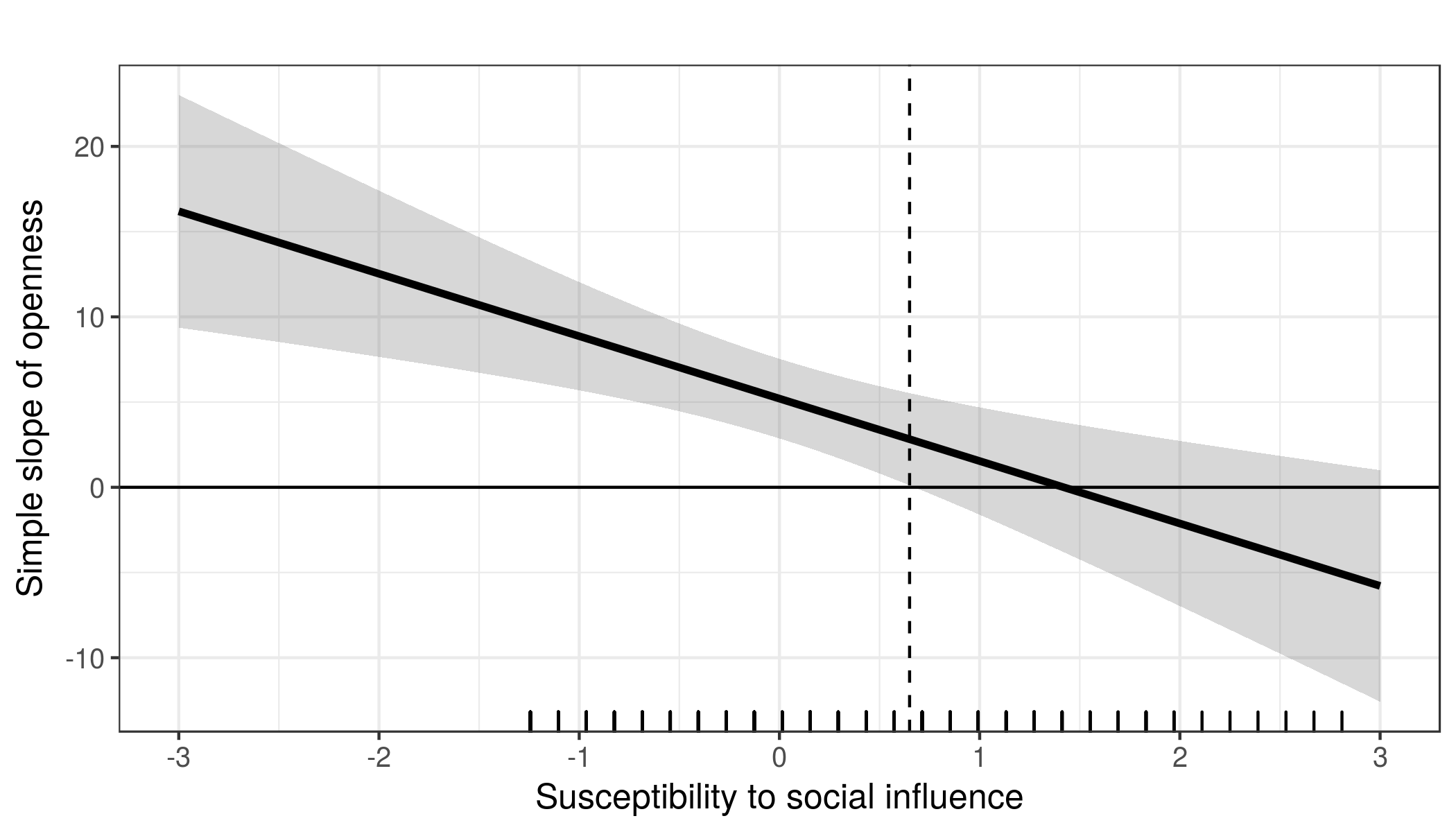


Visualization of the simple slope of openness on liking high-openness topics across the full range of SNI. The simple slope of openness on liking high-openness topics is significant in the interval [-3.00, 0.59] and positive when SNI is 0.7 SD above the mean and lower (71% of the observations of SNI are within this region). Note that the dashed vertical line indicates the level of the moderator at which openness becomes significantly associated with liking high-openness topics.

1. Note that all of the visualizations of interactions are based on recommendations and tools suggested by McCabe, Kim, and King (2018) (11). [↑](#footnote-ref-1)
