# Supplementary material for: Susceptibility to social influence predicts behavior on Facebook
Source: PLoS One. 2020 Mar 3;15(3):e0229337. doi: 10.1371/journal.pone.0229337 (PMC7053739; doi:10.1371/journal.pone.0229337)
Supplement: S3 Appendix — (DOCX) [file pone.0229337.s003.docx]

**SI Appendix 3: Additional details of Study 3**

Table S3.1: Queried Facebook likes that are indicative for the Big Five personality traits

|  | Extraversion | Openness | Conscientiousness | Agreeableness | Neuroticism |
| --- | --- | --- | --- | --- | --- |
| High | Beerpong  Michael Jordan  Dancing  Socializing  Chris Tucker  I Feel Better Tan  Modeling  Cheerleading  Theatre  Flip Cup | Oscar Wilde  Charles Bukowski  Sylvia Plath  Leonardo Da Vinci  Bauhaus  Dmt The Spirit Molecule  American Gods  John Waters  Plato  Leonard Cohen | Law Officer  National Law Enforcement Lowfares.Com  Accounting  Foursquare  Emergency Medical Services  Sunday Best  Kaplan University  Glock Inc  Mycalendar 2010 | Compassion International  Logan Utah  Jon Foreman  Redeeming Love  Pornography Harms  The Book Of Mormon  Circles Of Prayer  Go To Church  Christianity  Marianne Williamson | Sometimes I Hate Myself  Emo  Girl Interrupted  So So Happy  The Addams Family  Vocaloid  Sixbillionsecrets.com  Vampires Everywhere  Kurt Donald Cobain  Dot Dot Curve |
| Low | RPGs  Fanfiction.Net  Programming  Anime  Manga  Video Games  Role Playing Games  Minecraft  Voltaire  Terry Pratchet | NASCAR  Austin Collie  Monster-In-Law  I don’t read  Justin Moore  ESPN2  Farmlandia  The Bachelor  Oklahoma State University  Teen Mom 2 | Wes Anderson  Bandit Nation  Omegle  Vocaloid  Serial Killer  Screamo  Anime  Vamplets  Join If Ur Fat  Not Dying | I Hate Everyone  I Hate You  I Hate Police  Friedrich Nietzsche  Timmy South Park  Atheism / Satanism  Prada  Sun Tzu  Julius Caesar  Knives | Business Administration  Getting Money  Parkour  Track & Field  Skydiving  Mountain Biking  Soccer  Climbing  Physics / Engineering  48 Laws Of Power |

These 10 sets of Facebook topics are indicative of low and high expressions of the Big Five personality traits. We took the Facebook likes provided in the supplementary material of Kosinski et al., 2015. Vocaloid is a topic that is indicative of high neuroticism, as well as for low conscientiousness.

Table S3.2: Parameter estimates for the conditional effects and interactions of SNI and the Big Five personality traits on Facebook liking behavior

|  | *Β* | SE | 95% CI for *B* | *t* | *p* |
| --- | --- | --- | --- | --- | --- |
| Predicting likelihood of liking low-openness topics | | | | | |
| (Intercept) | 18.33 | 0.85 | [16.66, 20.01] | 21.50 | < 0.001 |
| Openness | -5.03 | 1.30 | [-7.58, -2.48] | -3.86 | **< 0.001** |
| SNI | 12.51 | 0.84 | [10.86, 14.16] | 14.90 | **< 0.001** |
| Openness*SNI | -3.79 | 1.20 | [-6.14, -1.45] | -3.17 | **< 0.01** |
| Predicting likelihood of liking high-openness topics | | | | | |
| (Intercept) | 23.68 | 0.95 | [21.81, 25.55] | 24.81 | < 0.001 |
| Openness | 3.22 | 1.46 | [0.35, -6.09] | 2.20 | < 0.05 |
| SNI | 11.56 | 0.94 | [9.72, 13.40] | 12.33 | **< 0.001** |
| Openness*SNI | -5.16 | 1.34 | [-7.79, -2.54] | -3.85 | **< 0.01** |
| Predicting likelihood of liking low-conscientiousness topics | | | | | |
| (Intercept) | 19.94 | 0.86 | [18.26, 21.62] | 23.23 | < 0.001 |
| Conscientiousness | -1.62 | 1.16 | [-3.88, 0.65] | -1.40 | 0.16 |
| SNI | 12.19 | 0.84 | [10.54, 13.83] | 14.52 | **< 0.001** |
| Conscientiousness*SNI | -2.82 | 1.11 | [-4.99, -0.65] | -2.55 | < 0.05 |
| Predicting likelihood of liking high-conscientiousness topics | | | | | |
| (Intercept) | 20.89 | 0.93 | [19.03, 22.69] | 22.33 | < 0.001 |
| Conscientiousness | 1.43 | 1.26 | [-1.03, 3.90] | 1.14 | 0.255 |
| SNI | 13.04 | 0.92 | [11.25, 14.83] | 14.25 | **< 0.001** |
| Conscientiousness*SNI | -2.23 | 1.21 | [-4.60, 0.14] | -1.85 | 0.065 |
| Predicting likelihood of liking low-extraversion topics | | | | | |
| (Intercept) | 30.29 | 0.98 | [28.37, 32.20] | 30.96 | < 0.001 |
| Extraversion | -0.56 | 1.18 | [-2.88, 1.76] | -0.47 | 0.636 |
| SNI | 11.41 | 0.95 | [9.54, 13.27] | 11.98 | **< 0.001** |
| Extraversion*SNI | -0.27 | 1.17 | [-2.55, 2.01] | -0.23 | 0.817 |
| Predicting likelihood of liking high-extraversion topics | | | | | |
| (Intercept) | 25.79 | 0.89 | [24.06, 27.53] | 29.13 | < 0.001 |
| Extraversion | 3.45 | 1.07 | [1.35, 5.55] | 3.22 | **0.001** |
| SNI | 12.85 | 0.86 | [11.16, 14.54] | 19.90 | **< 0.001** |
| Extraversion*SNI | 0.60 | 1.06 | [-1.47, 2.67] | 0.56 | 0.573 |
| Predicting likelihood of liking low-agreeableness topics | | | | | |
| (Intercept) | 21.79 | 0.81 | [20.20, 23.39] | 26.75 | < 0.001 |
| Agreeableness | -7.15 | 1.24 | [-9.57, -4.73] | -5.78 | **< 0.001** |
| SNI | 10.46 | 0.80 | [8.89, 12.03] | 13.02 | **< 0.001** |
| Agreeableness*SNI | -4.38 | 1.13 | [-6.60, -2.17] | -3.88 | **< 0.001** |
| Predicting likelihood of liking high-agreeableness topics | | | | | |
| (Intercept) | 18.44 | 0.84 | [16.79, 20.10] | 21.89 | < 0.001 |
| Agreeableness | -2.66 | 1.28 | [-5.16, -0.15] | -2.08 | < 0.05 |
| SNI | 12.44 | 0.83 | [10.81, 14.07] | 14.94 | **< 0.001** |
| Agreeableness*SNI | -6.54 | 1.17 | [-8.83, -4.24] | -5.58 | **< 0.001** |
| Predicting likelihood of liking low-neuroticism topics | | | | | |
| (Intercept) | 25.55 | 0.96 | [23.68, 27.43] | 26.68 | < 0.001 |
| Neuroticism | 5.67 | 1.83 | [2.08, 9.27] | 3.09 | **< 0.01** |
| SNI | 10.18 | 0.99 | [8.25, 12.12] | 10.32 | **< 0.001** |
| Neuroticism*SNI | 7.20 | 1.45 | [4.36, 10.04] | 4.97 | **< 0.001** |
| Predicting likelihood of liking high-neuroticism topics | | | | | |
| (Intercept) | 19.89 | 0.85 | [18.23, 21.56] | 23.46 | < 0.001 |
| Neuroticism | 12.75 | 1.62 | [9.58, 15.29] | 7.87 | **< 0.001** |
| SNI | 7.97 | 0.87 | [6.26, 9.68] | 9.12 | **< 0.001** |
| Neuroticism*SNI | 8.25 | 1.28 | [5.74, 10.76] | 6.44 | **< 0.001** |

Overall significance of the models: low openness: *F*(3, 534) = 105.10, *p* < 0.001, adjusted *R^2^* = 0.37 (6 missingness); high openness: *F*(3, 534) = 61.93, *p* < 0.001, adjusted *R^2^* = 0.25 (6 missingness); low conscientiousness: *F*(3, 534) = 86.46, *p* < 0.001, adjusted *R^2^* = 0.32 (6 missingness); high conscientiousness: *F*(3, 535) = 74.79, *p* < 0.001, adjusted *R^2^* = 0.29 (5 missingness); low-extraversion: *F*(3, 538) = 48.53, *p* < 0.001, adjusted *R^2^* = 0.21 (2 missingness); high extraversion: *F*(3, 537) = 84.19, *p* < 0.001, adjusted *R^2^* = 0.32 (3 missingness); low agreeableness: *F*(3, 533) = 92.25, *p* < 0.001, adjusted *R^2^* = 0.34 (7 missingness); high agreeableness: *F*(3, 534) = 105.40, *p* < 0.001, adjusted *R^2^* = 0.37 (6 missingness); low neuroticism: *F*(3, 538) = 84.72, *p* < 0.001, adjusted *R^2^* = 0.32 (2 missingness); high neuroticism: *F*(3, 535) = 123.80, *p* < 0.001, adjusted *R^2^* = 0.41 (5 missingness).

Note that the bold p values are also significant after Bonferroni correction.

Figure S3.3: SNI as a moderator of the effect of openness on liking low-openness topics


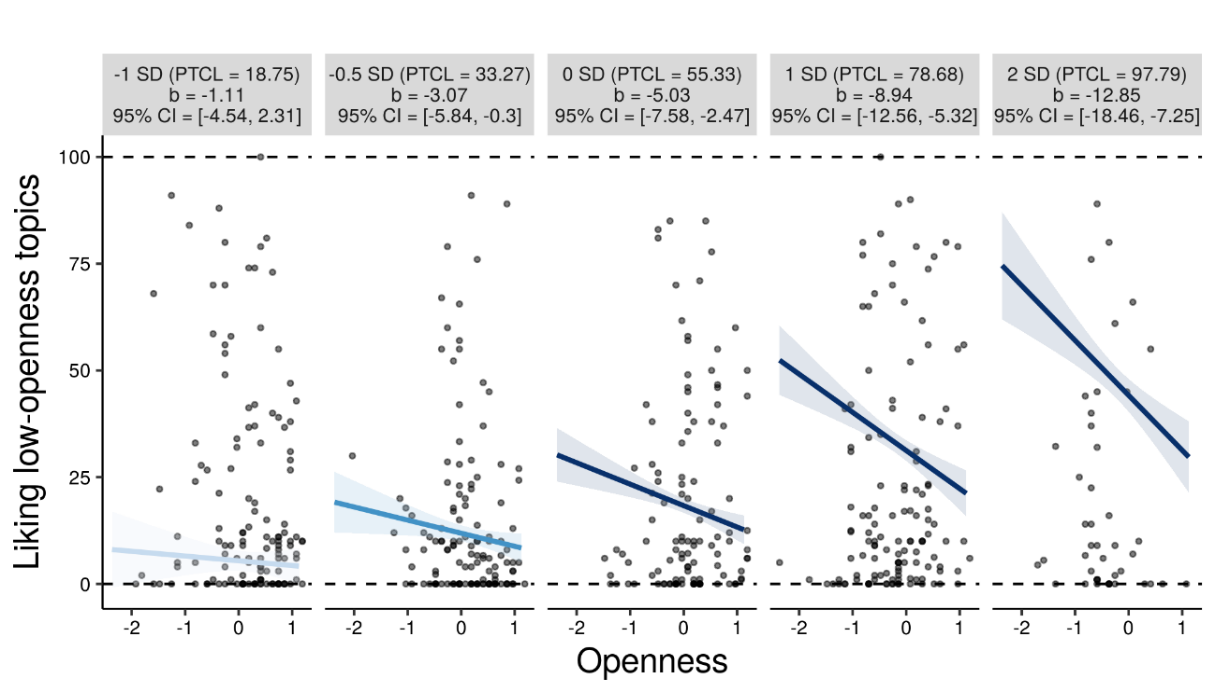


Simple slopes plot of the interaction of SNI and openness. The x axes represent openness (range of observed data for openness). Simple slopes are depicted for the levels of SNI, namely 1 SD and 0.5 SD below the mean as well as 1 SD and 2 SD above the mean (range of observed data for SNI). For all the slopes, we depict the 95% confidence region (shaded area), observed data (data points), and the maximum and minimum values of the response variable (dashed lines). CI = confidence interval, PTCL = percentile.

Figure S3.4: Johnson-Neyman plot (liking low-openness topics)


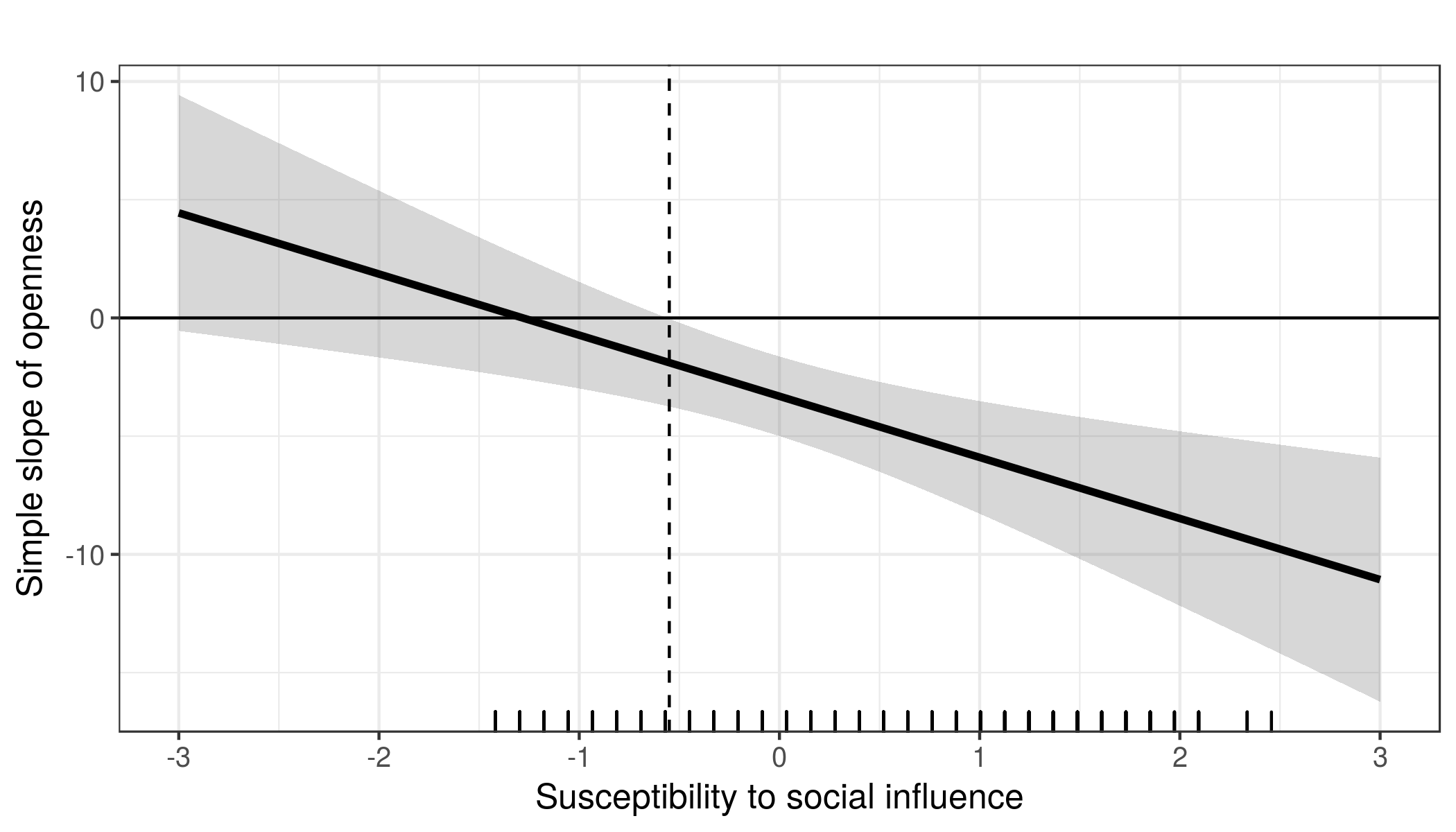


Visualization of the simple slope of openness on liking low-openness topics across the full range of SNI. The simple slope of openness on liking low-openness topics is significant in the interval [-0.59, 3.00] and negative when SNI is 0.6 SD below or further above the mean (67% of observations of SNI are within this region). The dashed vertical line indicates the level of SNI at which openness becomes significantly associated with liking low-openness topics.

Figure S3.5: SNI as a moderator of the effect of openness on liking high-openness topics


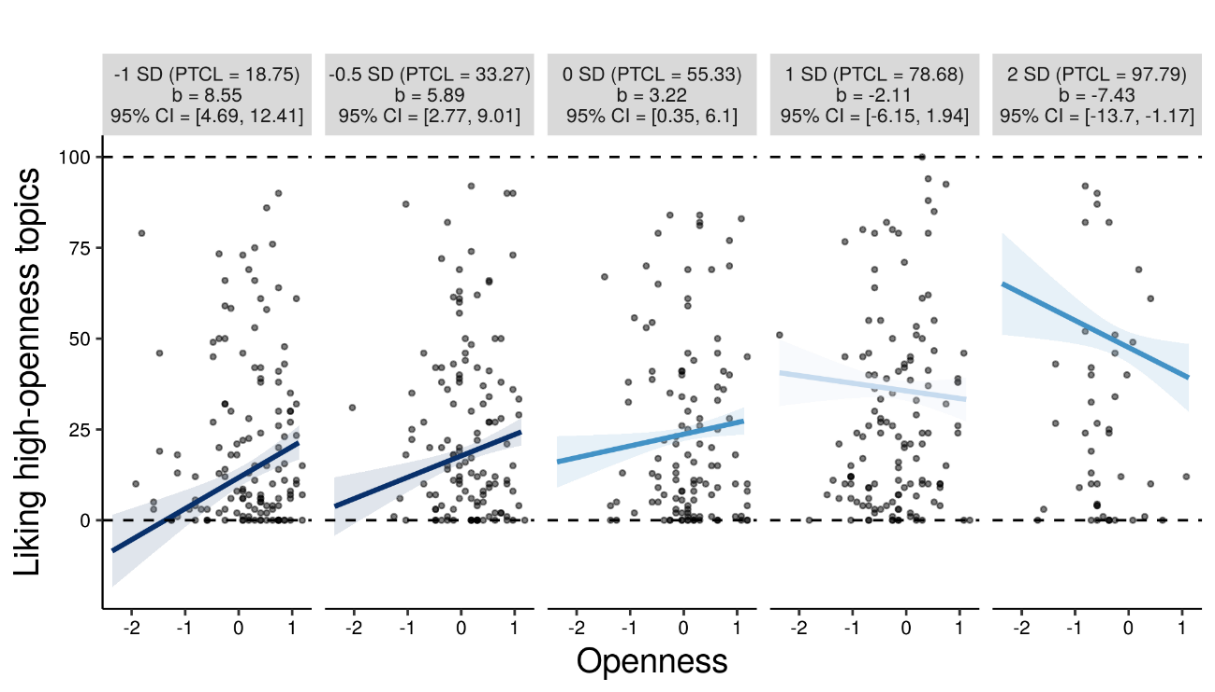


Simple slopes plot of the interaction of SNI and openness. The x axes represent openness (range of observed data for openness). Simple slopes are depicted for the levels of SNI, namely 1 SD and 0.5 SD below the mean as well as 1 SD and 2 SD above the mean (range of observed data for SNI). For all the slopes, we depict the 95% confidence region (shaded area), observed data (data points), and the maximum and minimum values of the response variable (dashed lines). CI = confidence interval, PTCL = percentile.

Figure S3.6: Johnson-Neyman plot (liking high-openness topics)


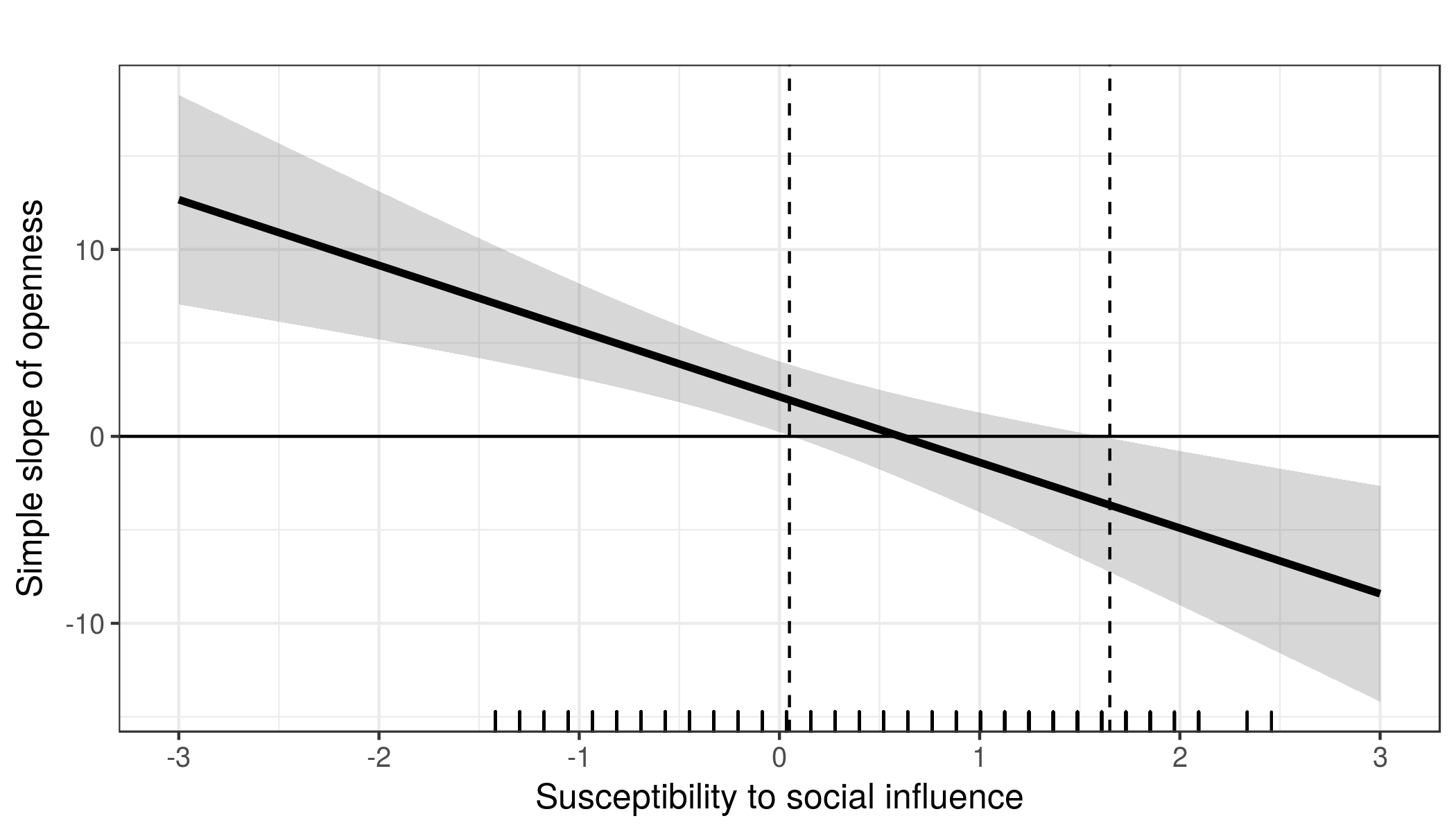


Visualization of the simple slope of openness on liking high-openness topics across the full range of SNI. The simple slope of openness on liking high-openness topics is significant in the interval [-3.00, 0.05] and [1.67, 3] and negative when SNI is 1.65 SD or further above the mean (7% of the observations of SNI are within this region). The simple slope of openness on liking high-openness topics is significantly positive when SNI is 0.05 SD above and further below the mean (59% of the observations of SNI are within this region).The dashed vertical lines indicate the levels of SNI at which openness becomes significantly associated with liking high-openness topics.

Figure S3.7: SNI as a moderator of the effect of agreeableness on liking low-agreeableness topics


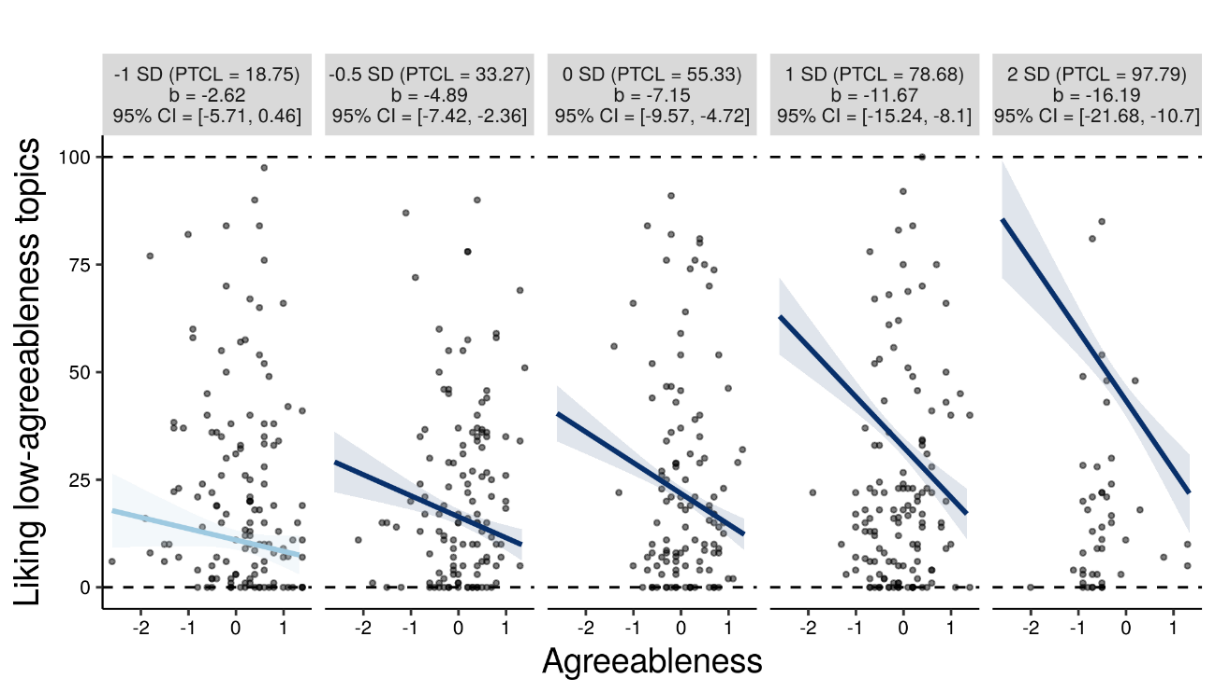


Simple slopes plot of the interaction of SNI and agreeableness. The x axes represent agreeableness (range of observed data for agreeableness). Simple slopes are depicted for levels of SNI, namely 1 SD and 0.5 SD below the mean as well as 1 SD and 2 SD above the mean (range of observed data for SNI). For all the slopes, we depict the 95% confidence region (shaded area), observed data (data points), and the maximum and minimum values of the response variable (dashed lines). CI = confidence interval, PTCL = percentile.

Figure S3.8: Johnson-Neyman plot (liking low-agreeableness topics)


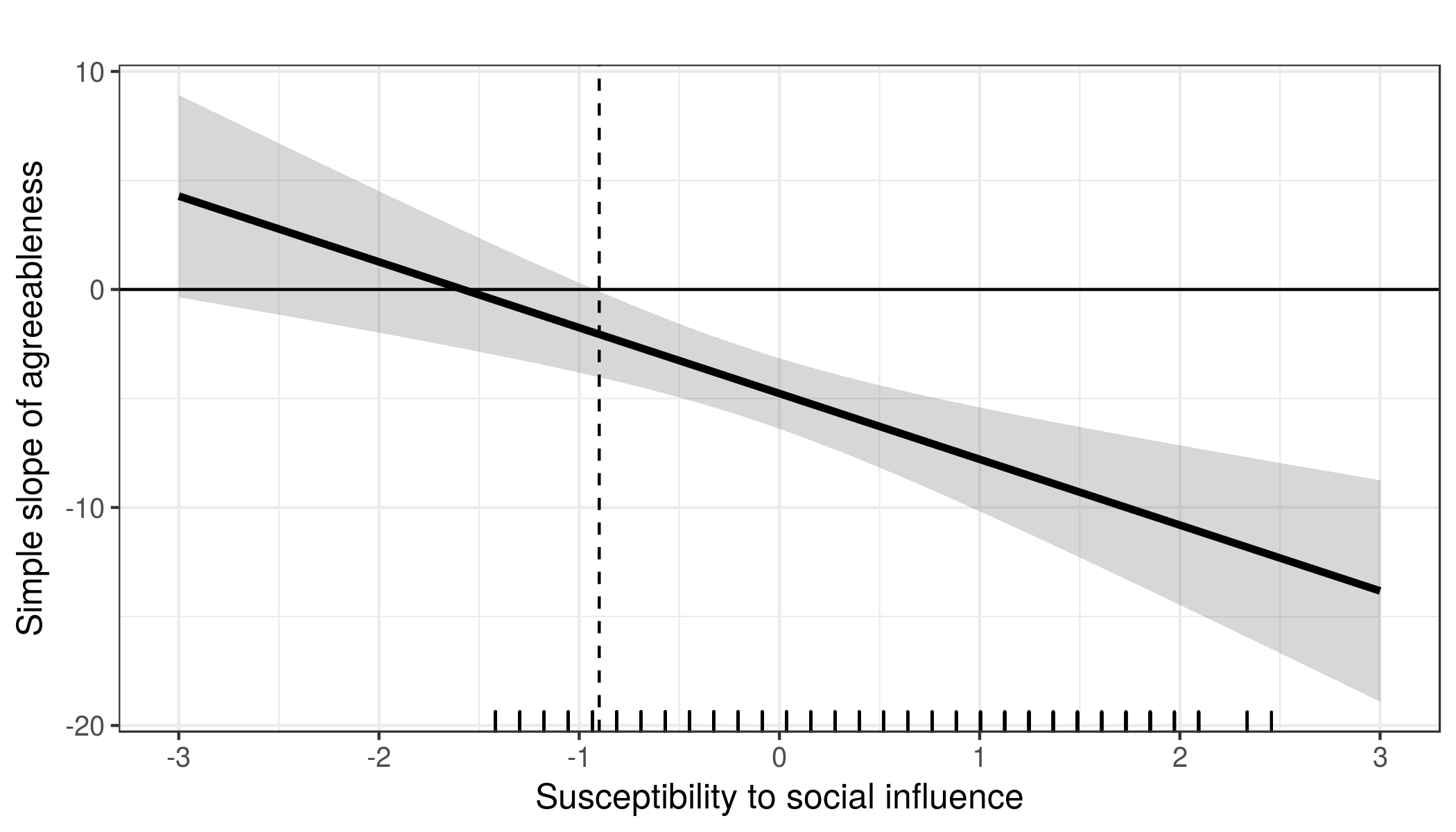


Visualization of the simple slope of agreeableness on liking low-agreeableness topics across the full range of SNI. The simple slope of agreeableness on liking low-agreeableness topics is significant in the interval [-3.00, -0.94] and negative when SNI is 0.9 SD below and further above the mean (79% of the observations of SNI are within this region). The dashed vertical line indicates the level of SNI at which agreeableness becomes significantly associated with liking low-agreeableness topics.

Figure S3.9: SNI as a moderator of the effect of agreeableness on liking high-agreeableness topics


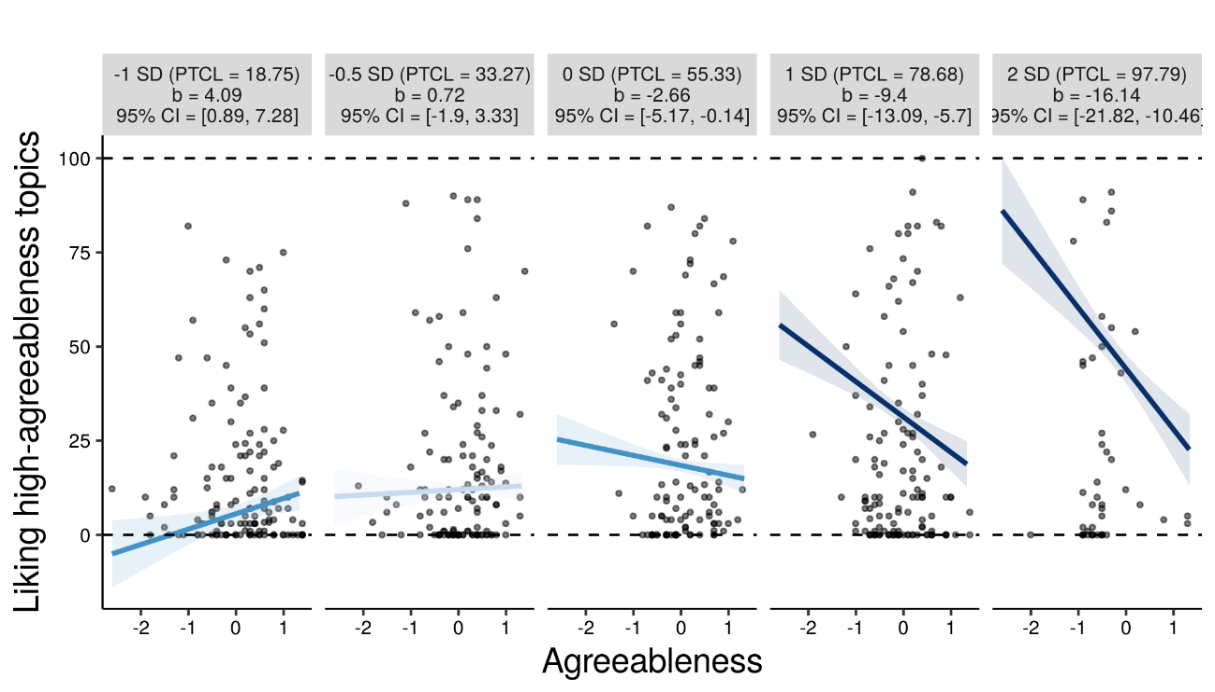


Simple slopes plot of the interaction of SNI and agreeableness. The x axes represent agreeableness (range of observed data for focal predictor). Simple slopes are depicted for the levels of SNI, namely 1 SD and 0.5 SD below the mean as well as 1 SD and 2 SD above the mean (range of observed data for SNI). For all the slopes, we depict the 95% confidence region (shaded area), observed data (data points), and the maximum and minimum values of the response variable (dashed lines). CI = confidence interval, PTCL = percentile.

Figure S3.10: Johnson-Neyman plot (liking high-agreeableness topics)


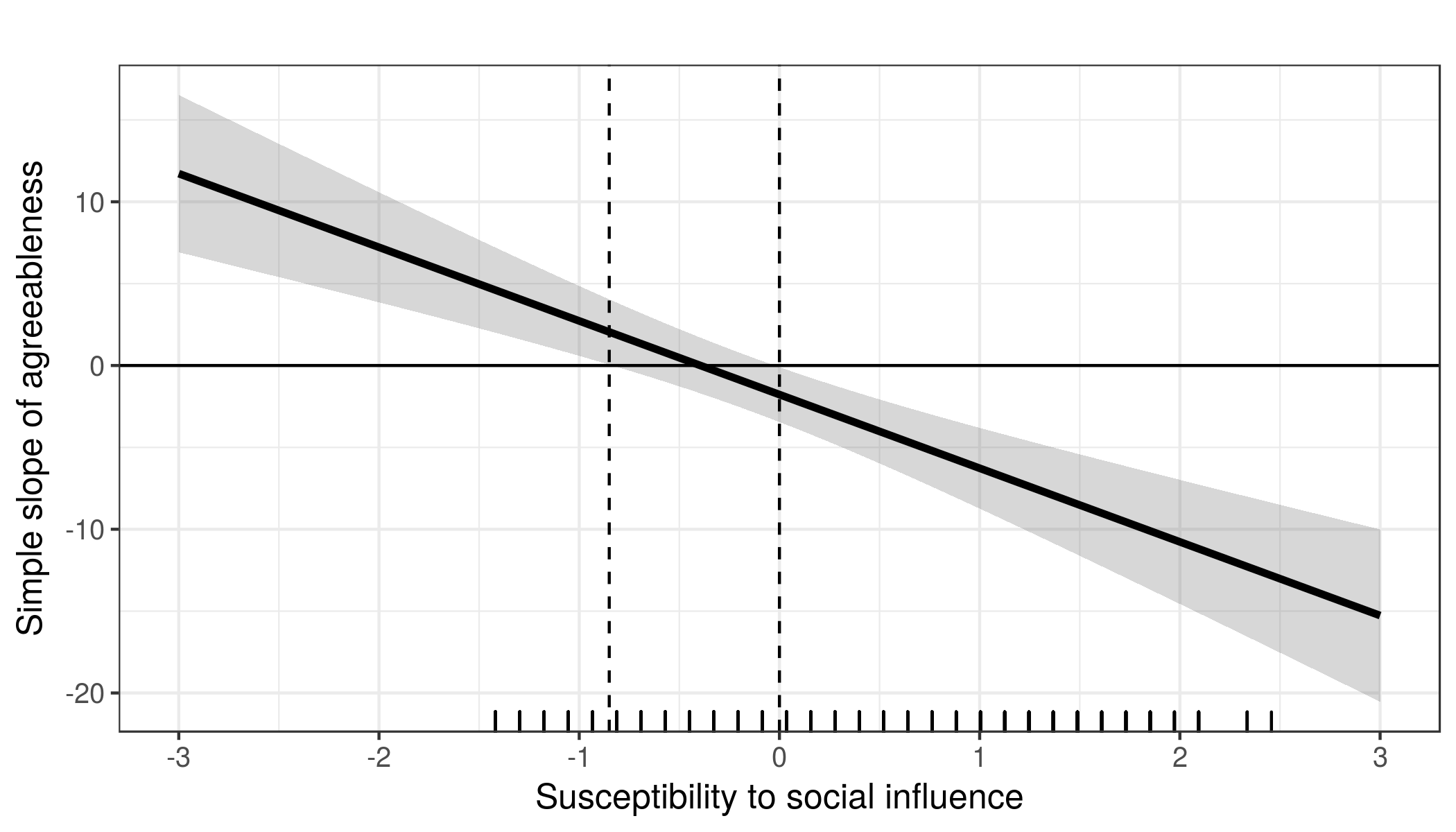


Visualization of the simple slope of agreeableness on liking high-agreeableness topics across the full range of SNI. The simple slope of agreeableness on liking high-agreeableness topics is significant outside the interval [-0.86, -0.02] and negative when SNI is around or above the mean (45% of the observations of SNI are within this region). The simple slope of agreeableness on liking high-agreeableness topics is significantly positive when SNI is 0.85 SD or further below the mean (21% of the observations of SNI are within this region). The dashed vertical lines indicate the levels of SNI at which agreeableness becomes significantly associated with liking high-agreeableness topics.

Figure S3.11: SNI as a moderator of the effect of neuroticism on liking low-neuroticism topics


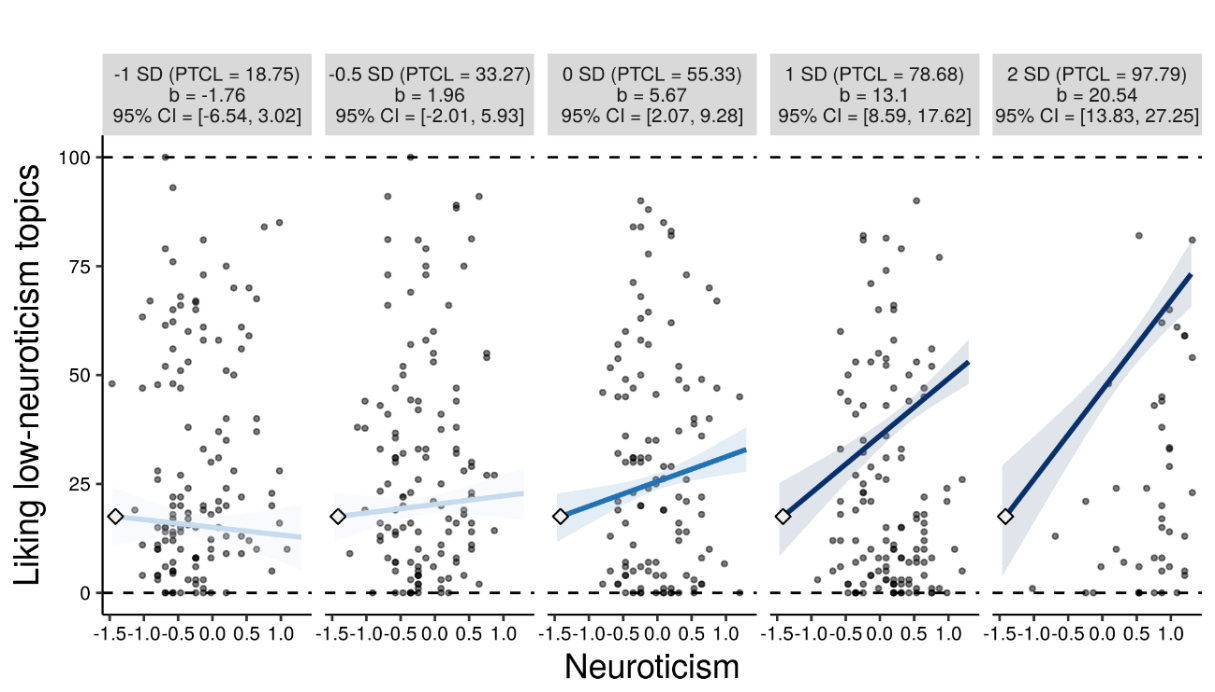


Simple slopes plot of the interaction of SNI and neuroticism. The x axes represent neuroticism (range of observed data for focal predictor). Simple slopes are depicted for the levels of SNI, namely 1 SD and 0.5 SD below the mean as well as 1 SD and 2 SD above the mean (range of observed data for SNI). For all the slopes, we depict the 95% confidence region (shaded area), observed data (data points), the maximum and minimum values of the response variable (dashed lines), and the crossover point (diamond). CI = confidence interval, PTCL = percentile.

Figure S3.12: Johnson-Neyman plot (liking low-neuroticism topics)


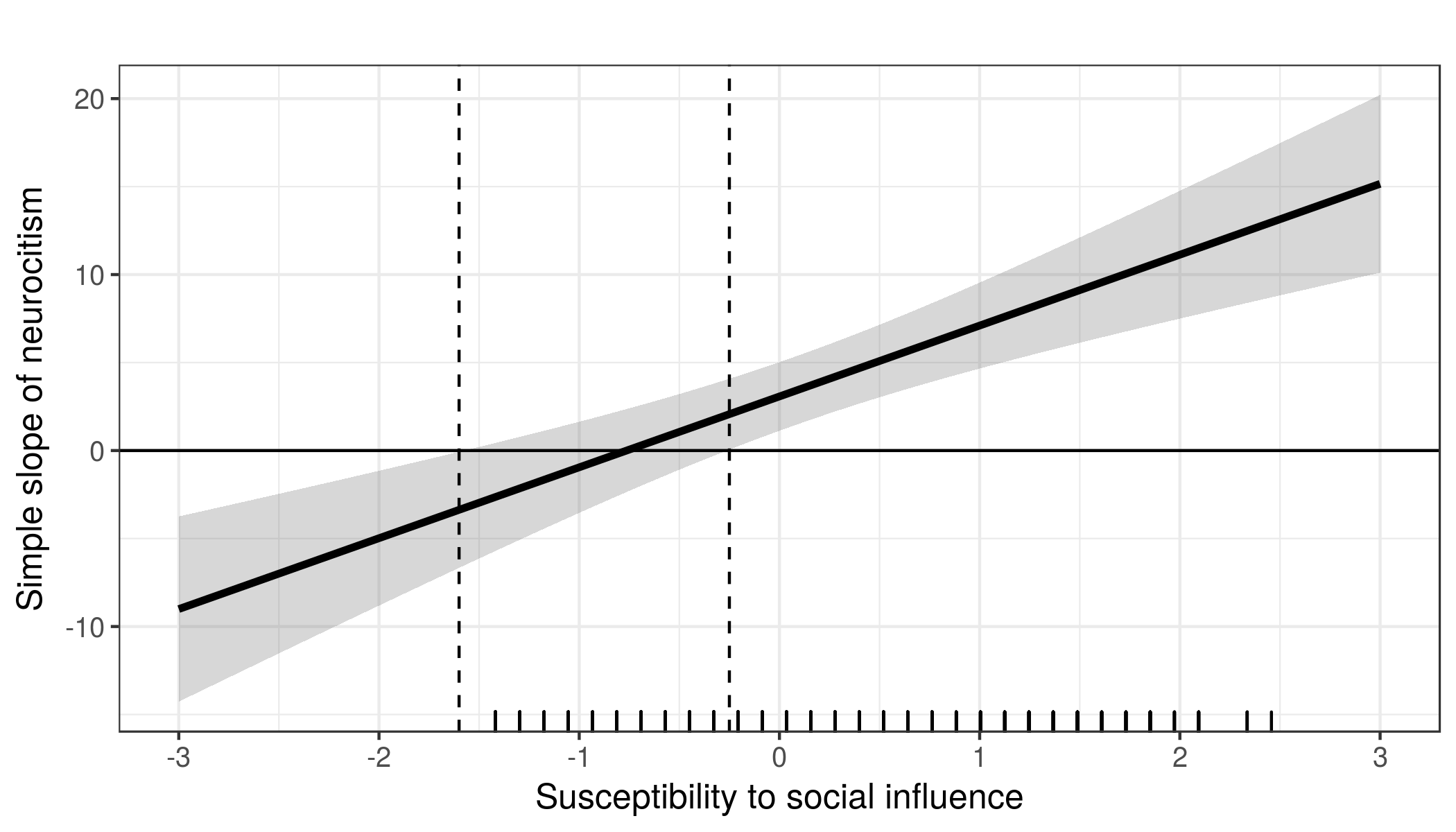


Visualization of the simple slope of neuroticism on liking low-neuroticism topics across the full range of SNI. The simple slope of neuroticism on liking low-neuroticism topics is significant in the interval [-1.63, -0.27] and negative when SNI is 1.6 SD or further below the mean (0% of the observations of SNI are within this region). The simple slope of neuroticism on liking high-neuroticism topics is significantly positive when SNI is 0.25 SD below or further above the mean (52.76% of the observations of SNI are within this region). The dashed vertical lines indicate the levels of SNI at which neuroticism becomes significantly associated with liking low-neuroticism topics.

Figure S3.13: SNI as a moderator of the effect of neuroticism on liking high-neuroticism topics


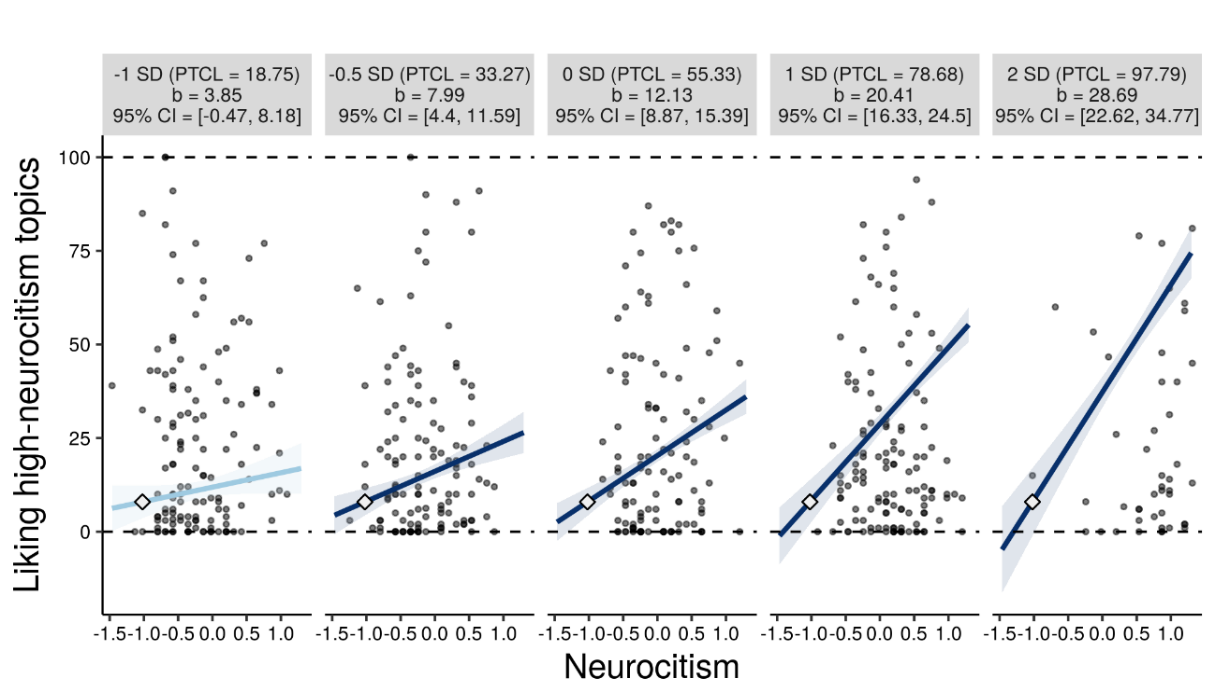


Simple slopes plot of the interaction of SNI and neuroticism. The x axes represent neuroticism (range of observed data for focal predictor). Simple slopes are depicted for the levels of SNI, namely 1 SD and 0.5 SD below the mean as well as 1 SD and 2 SD above the mean (range of observed data for SNI). For all the slopes, we depict the 95% confidence region (shaded area), observed data (data points), the maximum and minimum values of the response variable (dashed lines), and the crossover point (diamond). CI = confidence interval, PTCL = percentile.

Figure S3.14: Johnson-Neyman plot (liking high-neuroticism topics)


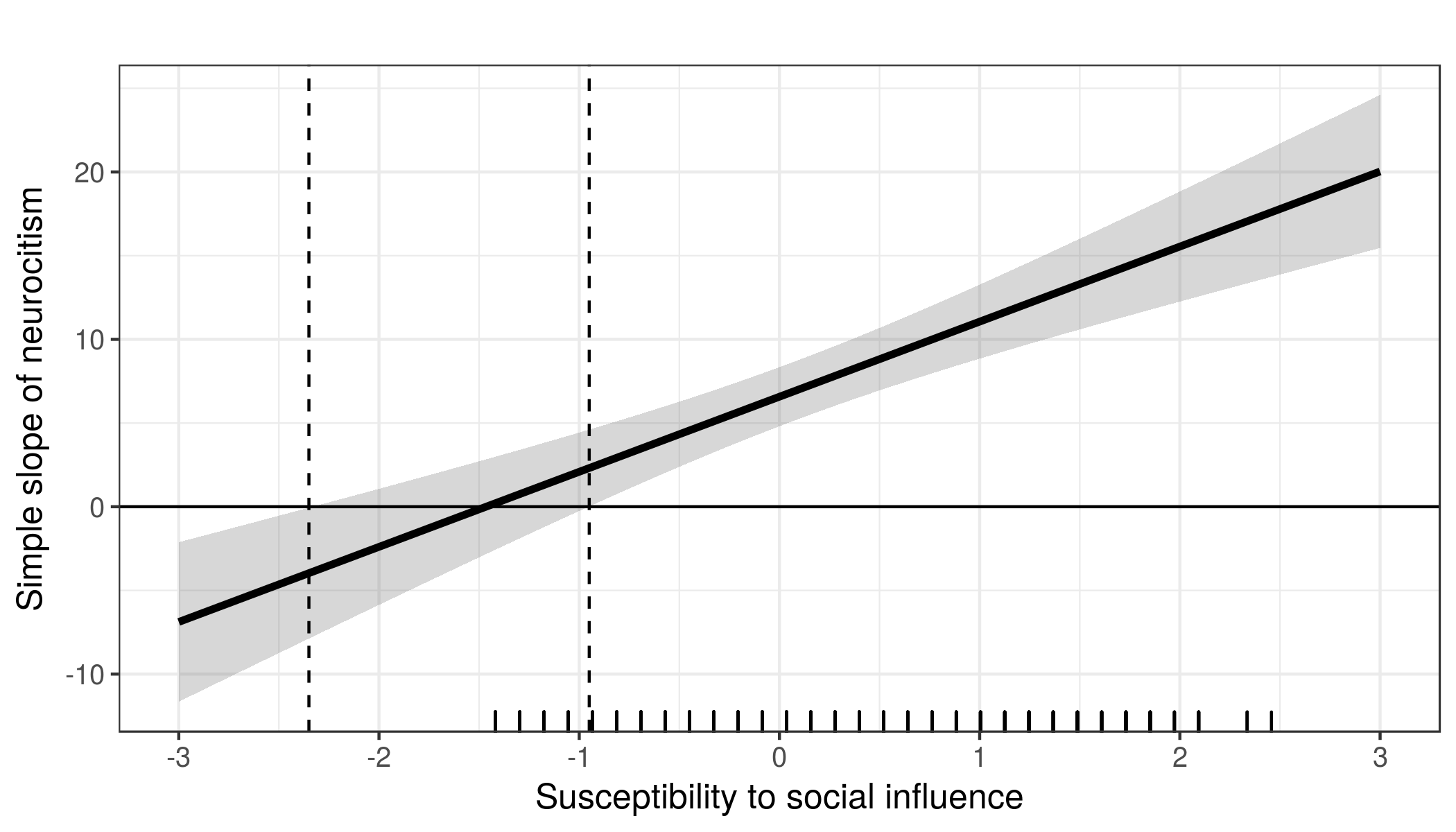


Visualization of the simple slope of neuroticism on liking high-neuroticism topics across the full range of SNI. The simple slope of neuroticism on liking high-neuroticism topics is significant outside the interval [-2.39, -1.03] and negative when SNI is 2.35 SD or further below the mean (0% of the observations of SNI are within this region). The simple slope of neuroticism on liking high- neuroticism topics is significantly positive when SNI is 0.95 SD below and further above the mean (81% of the observations of SNI are within this region). The dashed vertical lines indicate the levels of SNI at which neuroticism becomes significantly associated with liking high-neuroticism topics.

**References**

1. Bearden WO, Netemeyer RG, Teel JE. Measurement of consumer susceptibility to interpersonal influence. J Consum Res. 1989;15(4):473–481.

2. Bearden WO, Rose RL. Attention to social comparison information: An individual difference factor affecting consumer conformity. J Consum Res. 1990;16(4):461–471.

3. Reynolds FD, Darden WR. Mutually adaptive effects of interpersonal communication. J Mark Res. 1971;8(11):449–454.

4. Scheier MF, Carver CS. The self-consciousness scale: A Revised Version for Use with General Populations 1. J Appl Soc Psychol. 1985;15(8):687–699.

5. Cialdini RB, Trost MR, Newsom JT. Preference for consistency: The development of a valid measure and the discovery of surprising behavioral implications. J Pers Soc Psychol. 1995;69(2):318.

6. Obermiller C, Spangenberg ER. Development of a scale to measure consumer skepticism toward advertising. J Consum Psychol. 1998;7(2):159–186.

7. Tian KT, Bearden WO, Hunter GL. Consumers’ need for uniqueness: Scale development and validation. J Consum Res. 2001;28(1):50–66.

8. Bearden WO, Netemeyer RG. Handbook of marketing scales: Multi-item measures for marketing and consumer behavior research. Newbury Park, CA: Sage; 1999.

9. Boyle GJ, Saklofske DH, Matthews G. Measures of Personality and Social Psychological Constructs. London: Academic Press; 2014.

10. Bates D, Mächler M, Bolker B, Walker S. Fitting linear mixed-effects models using lme4. ArXiv Prepr ArXiv14065823. 2014.

11. McCabe CJ, Kim DS, King KM. Improving Present Practices in the Visual Display of Interactions. Adv Methods Pract Psychol Sci. 2018 Jun 1;1(2):147–165.
